# Creating true and false memories from forgotten information in *Drosophila*

**DOI:** 10.1038/s41593-026-02381-2

**Published:** 2026-07-30

**Authors:** Wenbin Yang, Benedetta Zattera, Miguel Pavão-Delgado, Carolin Warnecke, Johanna Aurelia Schweizer, Ana Ricciuti, Büşra Çoban, Johannes Felsenberg

**Affiliations:** 1 Friedrich Miescher Institute for Biomedical Research, Basel, Switzerland; 2 Faculty of Science, University of Basel, Basel, Switzerland

**Keywords:** Forgetting, Neural circuits

## Abstract

Recovering forgotten memories is a double-edged sword. While regaining access to forgotten information can be advantageous, targeted memory recovery carries the risk of generating false memories. The neuronal processes governing true memory recovery and false memory formation remain poorly understood. Here we show in *Drosophila* that reminder cues recover forgotten aversive memories. Forgotten memories persist as silent memory traces in behaviorally neutral mushroom body output neurons. From this silent state, reminders recover memory expression by reinstating the original memory trace in mushroom body output neurons that guide behavior. However, manipulating reminders can distort recovery, leading to false memory formation. The effectiveness of this distortion depends on whether misleading reminder cues were present during the original training event. Both true memory recovery and false memory formation are context-dependent processes gated by separable dopaminergic pathways. Together, these findings suggest that forgotten memories can re-emerge through a reconstructive process that flexibly integrates stored information and recent experiences.

## Abstract (web summary)

Forgotten memories in *Drosophila* persist as silent memory traces and can be recovered by reminder cues. Manipulating reminders can create false memories, showing that memory reconstruction flexibly integrates stored information with recent experiences.

## Main

Memory is fallible. Its accuracy can be reduced by a decrease in accessibility (forgetting) or by experience-dependent manipulation of the content^1,2^. While forgetting may permanently erase some memories, others can persist in an inaccessible state and can be recovered by reminder cues, implying that forgetting is reversible^3,4^. The recovery of forgotten memories depends on the reminder cues, making it susceptible to distortion by suggestive cues. In humans, suggestive questioning can implant detailed recollections of events that never occurred, including false witness statements or fabricated traumatic experiences^5–9^. False memories are not unique to humans and have been documented in other species^10–12^. However, little is known about the detailed neuronal processes underlying the correct and erroneous reconstruction of forgotten memories.

Studying memory and forgetting in *Drosophila melanogaster* allows for probing related neuronal circuits with cellular resolution^1,13^. Flies can learn to avoid an odor that predicts aversive electric shocks^14^. In the fly brain, learned information is stored in the mushroom body (MB)^15^. During aversive learning, activity of dopaminergic neurons (DANs) from the protocerebral posterior lateral cluster 1 (PPL1) drives plasticity between odor-coding Kenyon cells (KCs) and MB output neurons (MBONs)^16–20^. After learning, olfactory memory traces manifest as reduced activity in approach-coding MBONs, resulting in odor avoidance^18,21^. While the roles of approach-coding MBONs in storing and retrieving aversive memories are well established, the functions of many neutral MBONs that do not drive odor-guided approach or avoidance remain unknown^17,21,22^. Flies can learn from single-trial odor-shock pairing but memory expression decays rapidly within the first few hours, and 24 h after training the avoidance behavior is undetectable^14^. The molecular and cellular components that mediate such forgetting are well characterized^1,23–26^. During forgetting, the decay in learned behavior is paralleled, at least to a certain extent, by the decay of MBON memory traces^24,27^. Though recent studies suggest that after forgetting not all parts of memory are lost^26,28,29^, it remains unclear how forgotten memory can be recovered. In this study, we investigate the neuronal processes underlying the recovery of forgotten memories and the formation of false memories.

## Results

### Reminders can recover forgotten aversive memories

**Fig. 1:**
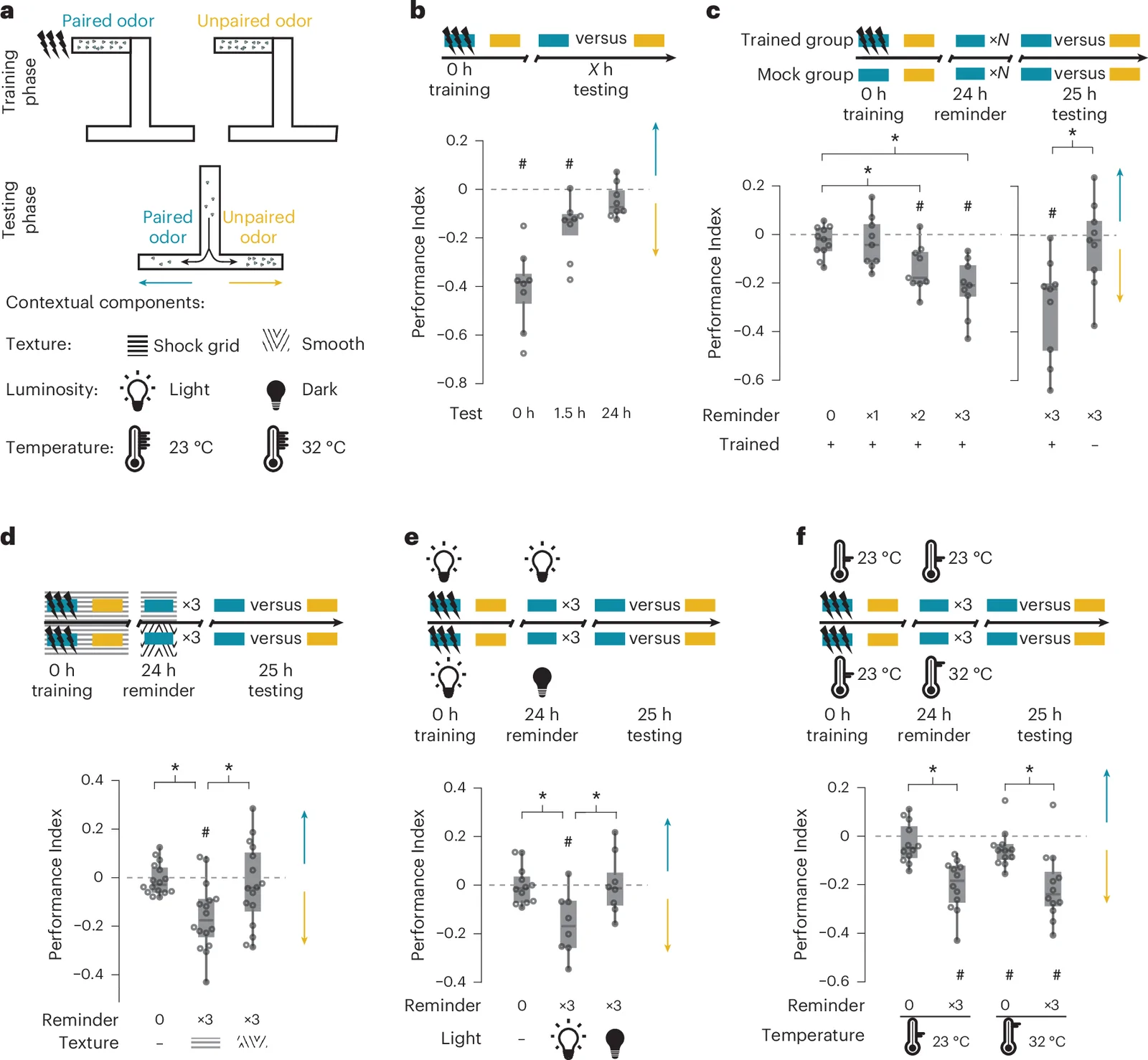
Context-dependent reminders recover forgotten memories. **a**, Top: illustration of experimental setting for T-maze training. Colors indicate the paired odor (blue) and the unpaired odor (yellow). Colored arrows represent flies’ approach toward the paired (blue arrow) or unpaired (yellow arrow) odor. Bottom: illustration of contextual components tested in **d**, **e** and **f**. **b**, Top: protocol. Bottom: single-trial aversive memory expression decays over time. For each group *n* = 8. **c**, Top: protocols. Bottom: repeated reminders recover forgotten memory in trained but not mock-trained flies (right side of graph). 0 × reminder/trained: *n* = 11, 1 × reminder/trained: *n* = 9, 2 × reminder/trained: *n* = 9, 3 × reminder/trained: *n* = 9, 3 × reminder/trained: *n* = 9, 3 × reminder/mock: *n* = 9. **d**, Top: protocols indicate contextual changes between training and reminders. Bottom: memory recovery is context-dependent; changed texture during reminders prevents recovery. For each group *n* = 16. **e**, Top: protocols. Bottom: changed lighting conditions during reminders prevent recovery. No reminder: *n* = 12, reminder in training light: *n* = 8, reminder in darkness: *n* = 8. **f**, Top: protocols. Bottom: changing temperature during reminders does not prevent recovery. For each group *n* = 12. Asterisk (*) indicates significant difference; hash (#) indicates difference from zero. All statistical tests and *P* values are reported in Supplementary Table 2. Data are presented as box and whisker plots showing the median, interquartile range (IQR) and whiskers extending to the most extreme values within 1.5× IQR. Source data

**Extended Data Fig. 1:**
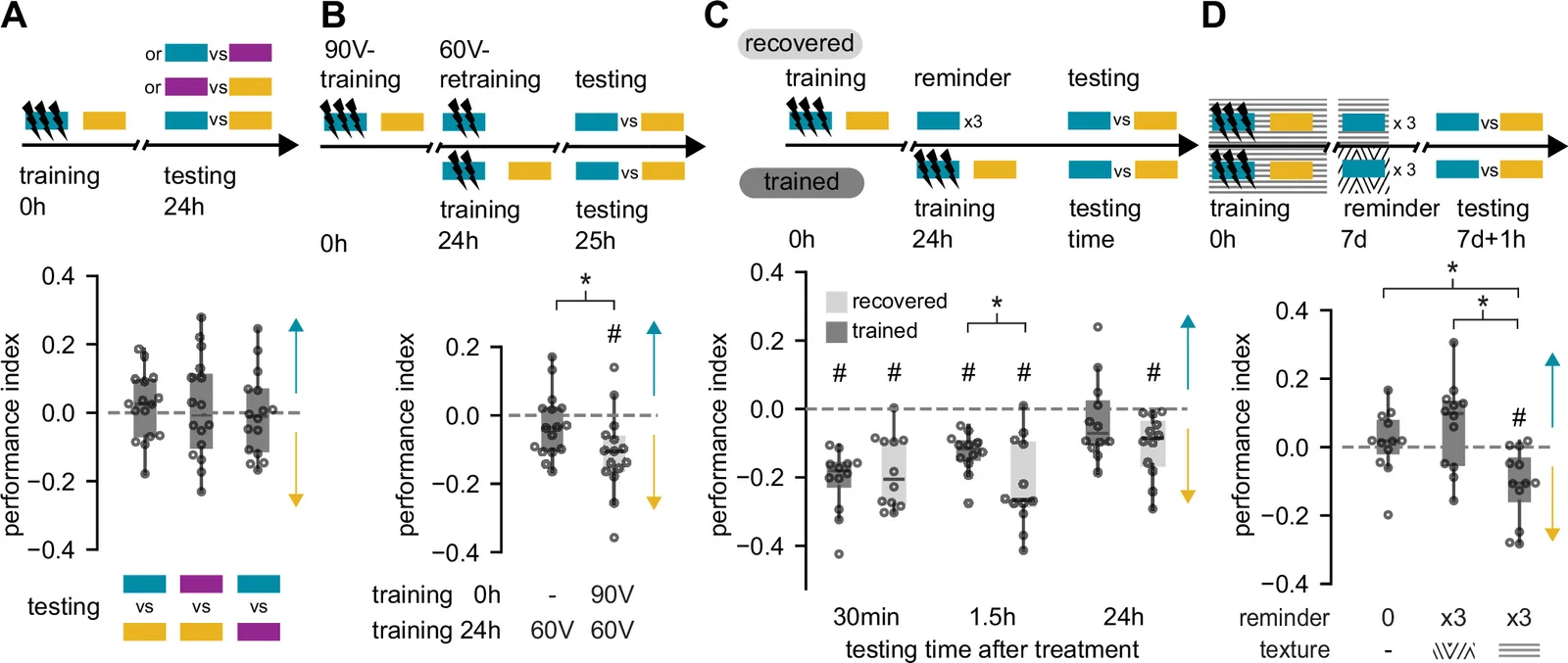
Context-dependent reminders recover forgotten memories. **a**) Top: protocol. Bottom: no aversive memory found at 24 h for either paired odor or unpaired odor. For each group n = 16. **b**) Top: protocol. Bottom: weak 60 V training fails to induce 1-h memory but retraining with 60 V recovers aversive memory. For each group n = 16. **c**) Top: protocol. Bottom: recovered memory appears more persistent than initial single-trial memory. For each group n = 12. **d**) Top: protocol. Bottom: reminders can recover forgotten memory one week after training. For each group n = 12. In all panels, asterisks denote a significant difference between groups and hash indicates difference from zero. All statistical tests and *p values* are reported in Supplementary Table 2. Data are presented as box-and-whisker plots showing the median, interquartile range, and whiskers extending to the most extreme values within 1.5xIQR. Source data

To investigate the reversibility of forgetting, we used single-trial aversive olfactory conditioning. In T-maze training chambers, flies were exposed to a first odor (paired odor) together with electric shocks for 1 min, followed by 45 s of clean air, and then exposed to a second odor (unpaired odor) for 1 min (ref. ^14^) (Fig. 1a). To reliably examine forgetting, we established training conditions that generated mild aversive memories. Testing learned avoidance at various time points after training revealed that learned avoidance decayed rapidly within the first hours and was undetectable after 24 h (Methods; Fig. 1b and Extended Data Fig. 1a).

To explore the recoverability of forgotten memories, we tested whether reminders resembling the training conditions reinstate learned avoidance. Indeed, re-exposing trained flies to the paired odor in the training chamber for two or three 1-min trials without electric shocks recovered the forgotten memory (Fig. 1c). Importantly, applying the three-trial recovery protocol to mock-trained flies (no electric shocks during training) did not elicit avoidance behavior (Fig. 1c). Likewise, retraining flies with a weak-conditioning protocol that alone does not induce detectable aversive memory reinstated learned avoidance, indicating that memory recovery is robust across distinct protocols (Extended Data Fig. 1b). Furthermore, directly comparing recovered memory with the initial memory revealed that the three-trial reminder protocol fully reinstated the learned avoidance (Extended Data Fig. 1c). Interestingly, the recovered memories, though subject to decay, appeared more persistent compared to the initial memory (Extended Data Fig. 1c).

Investigating the conditions necessary for memory recovery, we found that the effectiveness of reminders depends on contextual cues. Altering the lighting conditions or removing the chamber’s electric grid during the reminder trials prevented the recovery of learned avoidance behavior (Fig. 1a,d,e). By contrast, changing the temperature during reminder trials did not impair the recovery of forgotten memories (Fig. 1f). These findings indicate that contextual information, in addition to the paired odor, is critical to recovering the forgotten memory.

Collectively, our results demonstrate that context-dependent reminders can recover forgotten aversive memory. Notably, presenting reminders one week after training can still reinstate learned avoidance (Extended Data Fig. 1d). This suggests that a long-lasting memory persists in a silent state, which does not support memory expression. Consistent with previous studies^30^, we refer to this recoverable but unexpressed memory as a silent memory.

### A single pair of dopaminergic neurons gates the recovery of forgotten memory

**Fig. 2:**
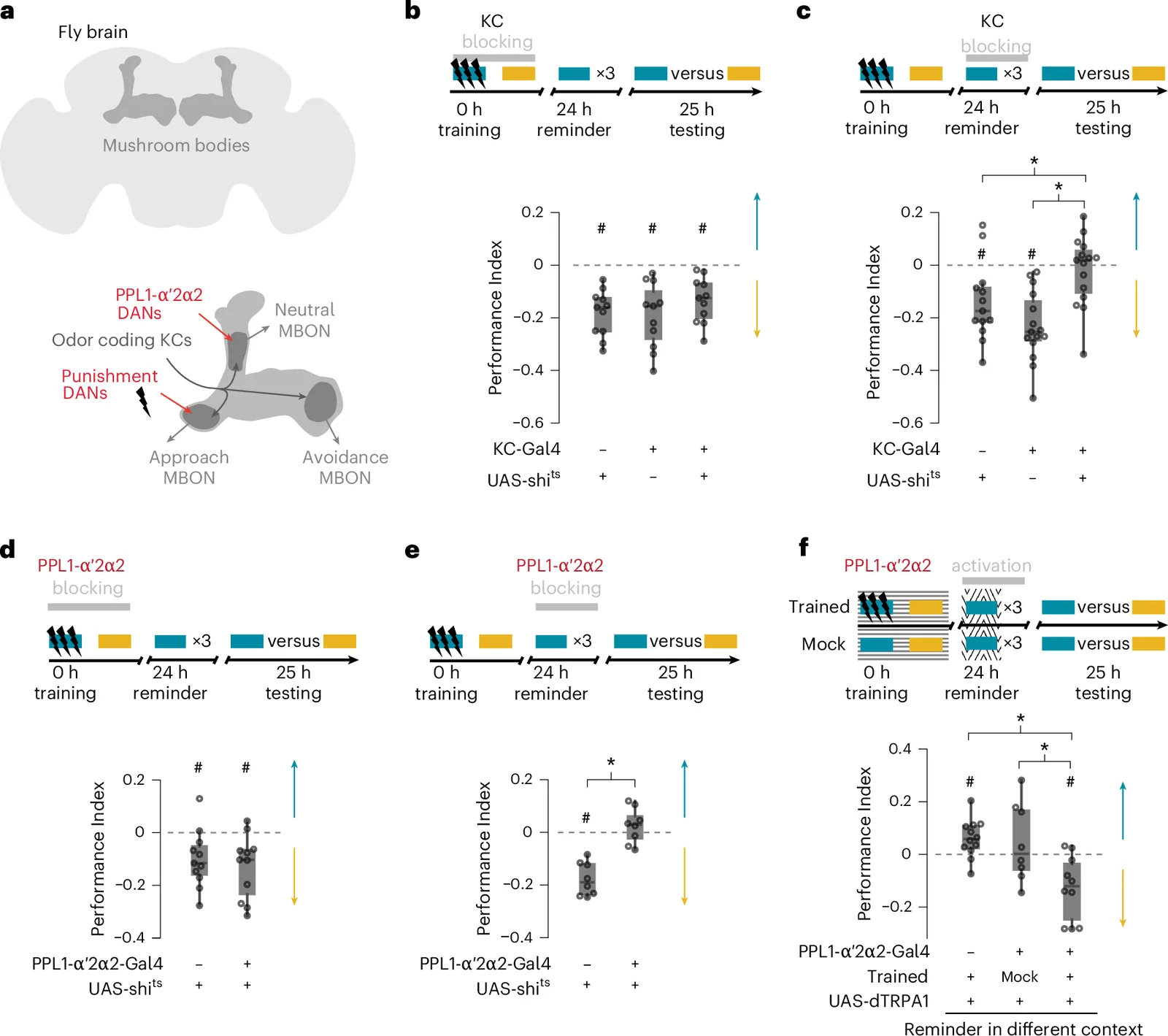
A single pair of dopaminergic neurons supports recovery of forgotten memory. **a**, Illustrations of a fly brain and MB. **b**, Top: protocol. Bottom: blocking KCs during training does not affect recovery of learned avoidance. For each group *n* = 11. **c**, Top: protocol. Bottom: blocking KCs during reminders prevents memory recovery. wild-type/Shi: *n* = 13, R13F02/wild-type: *n* = 15, R13F02/Shi: *n* = 15. **d**, Top: protocol. Bottom: blocking PPL1-α‘2α2 recovery DANs during training does not affect recovery. For each group *n* = 11. **e**, Top: protocol. Bottom: blocking PPL1-α‘2α2 recovery DANs during reminders prevents memory recovery. For each group *n* = 8. **f**, Top: protocol. Bottom: activating PPL1-α‘2α2 recovery DANs during reminder presentations in a different context is sufficient for memory recovery. Mock-trained flies do not show paired odor avoidance. Empty split-Gal4/Trp + training: *n* = 12, MB058B/Trp + mock: *n* = 8, MB058B/Trp + training: *n* = 10. Asterisk (*) indicates significant difference; hash (#) indicates difference from zero. All statistical tests and *P* values are reported in Supplementary Table 2. Data are presented as box and whisker plots showing the median, IQR and whiskers extending to the most extreme values within 1.5× IQR. Gal4-drivers: R13F02 for KCs, MB058B for PPL1-α‘2α2 and empty-Gal4 for PPL1-α‘2α2 genetic control. Source data

**Extended Data Fig. 2:**
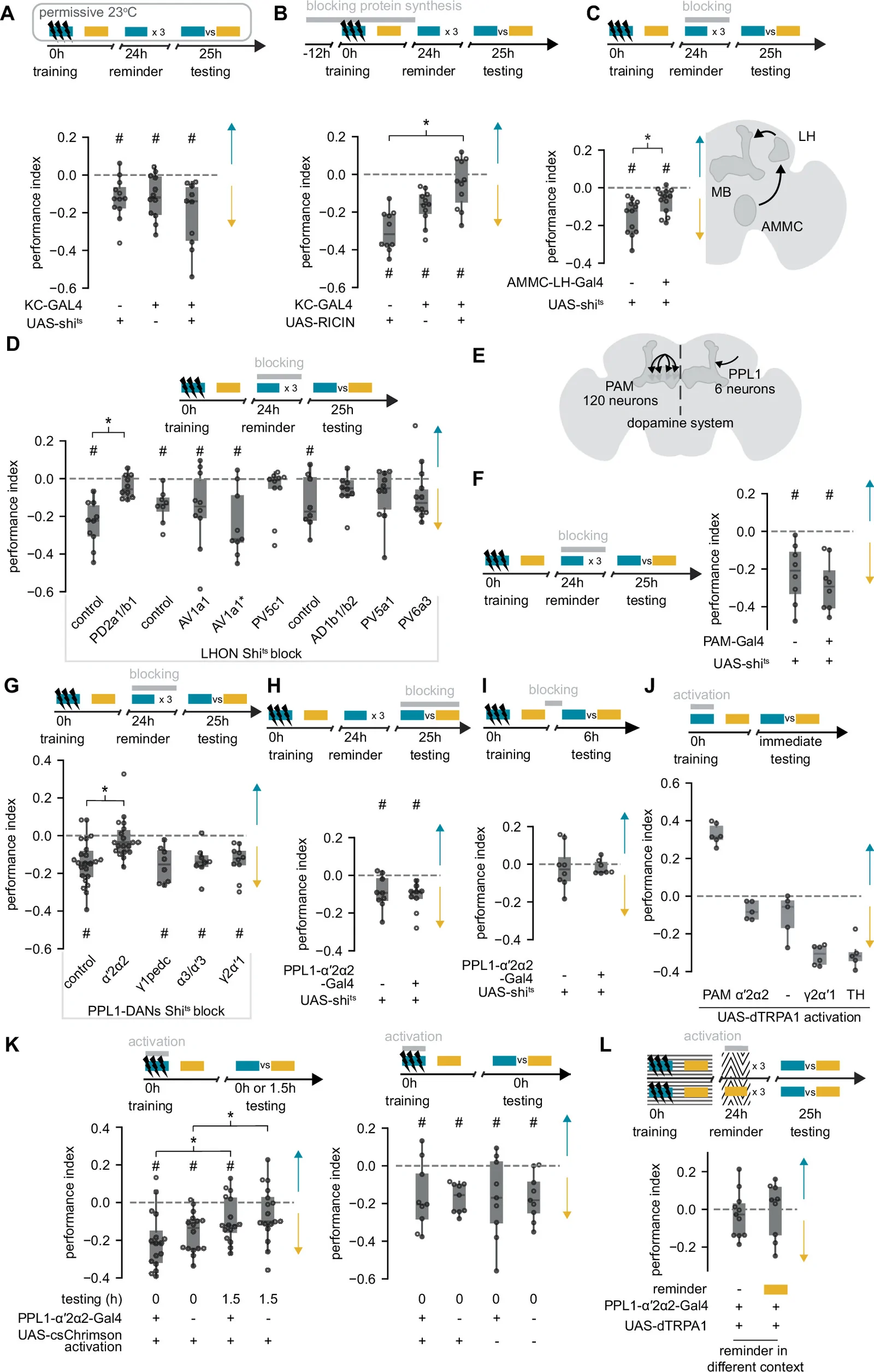
A single pair of dopaminergic neurons gates the recovery of forgotten memory. **a**) Top: protocol. Bottom: temperature control experiment from Fig. 2b and c; all flies recover learned avoidance. CS/Shi n = 12, R13F02/CS n = 12, R13F02/Shi n = 10. **b**) Top: protocol. Bottom: blocking protein synthesis during training until reminder session prevents recovery of learned avoidance. CS/RICIN n = 10, R13F02/CS n = 11, R13F02/RICIN n = 11. **c**) Top: protocol. Bottom left: blocking AMMC neurons during reminders prevents memory recovery. Bottom right: half-brain illustration of information flow between AMMC, lateral horn and mushroom body. Empty split-Gal4/Shi n = 12, NP1004/Shi n = 13. **d**) Top: protocol. Bottom: blocking lateral horn output neurons PD2a1/b1 neurons during reminders prevents memory recovery. Empty split-Gal4/Shi n = 10, LH989/Shi n = 10, empty split-Gal4/Shi n = 8, LH1983/Shi n = 10, LH728/Shi n = 9, LH1293/Shi n = 10, empty split-Gal4/Shi n = 8, LH1538/Shi n = 10, LH291/Shi n = 10, LH2193/Shi n = 10. **e**) Illustration of the mushroom body dopaminergic system. **f**) Top: protocol. Bottom: blocking PAM dopaminergic neurons during reminders leaves memory recovery intact. For each group n = 8. **g**) Top: protocol. Bottom: Blocking PPL1-α‘2α2 neurons during reminders prevents memory recovery. Empty split-Gal4/Shi n = 23, MB058B/Shi n = 19, MB320C/Shi n = 8, MB630B/Shi n = 8, MB296B/Shi n = 10. **h**) Top: protocol. Bottom: Blocking PPL1-α‘2α2 neurons during test does not affect retrieval of recovered memory. For each group n = 8. **i**) Top: protocol. Bottom: Blocking PPL1-α‘2α2 neurons after training does not affect forgetting. For each group n = 9. **j**) Top: protocol. Bottom: immediate memory test after training with artificial activation of different dopaminergic neurons. R58E02/Trp n = 6, MB058B/Trp n = 5, CS/Trp n = 5, MB296B/Trp n = 6, TH/Trp n = 5. **k**) Top: protocol. Bottom: optogenetic activation of recovery DANs during paired odor training does not affect learning nor extend time of forgetting. For each group of the first plot n = 17. For each group of the second plot n = 16. **l**) Top: protocol. Bottom: PPL1-α′2α2 activation during unpaired odor or no-odor reminder, does not recover learned avoidance. MB058B-Gal4/trp – no reminder n = 11, MB058B-Gal4/trp unpaired odor n = 9. In all panels, asterisks indicate significant differences between groups and hash significant differences from zero. All statistical tests and p values are reported in Supplementary Table 2. Data are presented as box-and-whisker plots showing the median, interquartile range, and whiskers extending to the most extreme values within 1.5xIQR. Gal4-drivers: R58E02-Gal4 for PAM-Gal4, MB058B-Gal4 for PPL1-α′2α2, MB296B-Gal4 for PPL1-γ2α’1, MB630B for PPL1-α3/α‘3 and empty-Gal4 for genetic control in all panels except 2 A and 2B. Source data

We found that recovery of forgotten memory required both the paired odor and the training context as a reminder. Olfactory memories are stored in the MB (Fig. 2a), and their retrieval requires output from KCs^15,31^. To investigate the functional role of specific MB circuits in the recovery of forgotten memory, we used the temperature-sensitive dynamin transgene *Shibire*^*ts1*^ (*Shi*^*ts1*^)^32^. Expressing *Shi*^*ts1*^ allows for the selective blockade of vesicle release from targeted neurons during either the training or the reminder sessions. Although KC to KC output is required to maintain sparse odor representations^33^, when KC output was blocked during training, memory recovery remained intact (Fig. 2b). By contrast, blocking KC output during the reminder session abolished memory recovery (Fig. 2c). Importantly, when the entire experiment was conducted at the permissive temperature, forgotten memory was recovered across all groups (Extended Data Fig. 2a). These findings indicate that the recovery of forgotten memories relies on a persistent silent memory that is dependent on KC output. Consistent with the notion that persistent memories require protein synthesis, we found that blocking protein synthesis in KCs during and after training using the cold-sensitive mutation of ricin toxin (ricin^CS^) prevented the recovery of learned avoidance (Extended Data Fig. 2b)^34,35^.

Though little is known about how contextual information is stored in the fly brain, previous work has suggested that circuits within the lateral horn (LH), a multisensory neuropil receiving olfactory, mechanosensory, visual and gustatory input, can store contextual information^28,36^. Consistent with this idea, we found that a mechanosensory pathway from the antennal mechanosensory and motor center to the LH, previously implicated in detecting the texture component of the context^28^, is required for the recovery of forgotten memory (Extended Data Fig. 2c). To further explore the role of the LH, we tested whether selected valence-coding LH output neurons (LHONs) contribute to memory recovery^37,38^. Silencing other LHONs produced either partial reductions in recovery or no detectable effect (Extended Data Fig. 2d). These results indicate that distributed LH output pathways contribute to reinstating forgotten aversive memory. Notably, PD2a1/b1 LHONs converge with MB circuits, suggesting that they integrate contextual information represented in the LH with memory-related signals stored in the MB^38,39^.

Within the MB, the dopaminergic system is critical in assigning valence to odors^13,19,20^ (Extended Data Fig. 2e). We therefore investigated whether the DANs innervating the MB contribute to the recovery of forgotten memories. Using *Shi*^*ts1*^, we selectively blocked vesicle release from specific DANs during the reminder sessions. Screening all reward- and punishment-coding DANs revealed that vesicle release exclusively from the two PPL1-α′2α2 DANs is required for recovering learned avoidance behavior (Fig. 2e and Extended Data Fig. 2f,g). In contrast, blocking PPL1-α′2α2 DANs either during training or during the test failed to affect either memory recovery or its expression (Fig. 2d and Extended Data Fig. 2h). In addition, blocking PPL1-α′2α2 neurons between training and testing left forgetting unchanged (Extended Data Fig. 2i). Together, these data show that PPL1-α′2α2 neurons are selectively engaged during reminders to recover forgotten memory. Accordingly, we refer to the two PPL1-α′2α2 neurons as the recovery DANs.

Artificial activation of specific PPL1 DANs that respond to electric shock has been shown to substitute for aversive teaching signals^20^. However, previous studies indicated that recovery DANs respond minimally to electric shock and lack the capacity to assign negative valence to odors^20,40^. Using the *dTrpA1*-encoded transient receptor potential (TRP) channel that depolarizes neurons at temperatures above 25 °C (ref. ^41^), we confirmed that thermogenetic activation of recovery DANs cannot substitute for aversive stimuli (Extended Data Fig. 2j). Further, using the red-shifted channelrhodopsins Chrimson^42^ we showed that activating recovery DANs during training did not facilitate aversive learning (Extended Data Fig. 2k).

In addition to providing teaching signals, DANs can provide state-dependent control of memory retrieval^43^. To test whether artificial activation of recovery DANs could support memory recovery under insufficient conditions, such as the absence of contextual information, we trained flies or exposed them to mock training. One day later, all groups were exposed to insufficient reminder sessions (different context). Thermogenetic activation of recovery DANs during the reminders in mock-trained flies did not induce avoidance, confirming that pairing an odor with recovery DAN activity does not lead to aversive memory formation (Fig. 2f). By contrast, activating recovery DANs during reminders in trained flies restored learned avoidance (Fig. 2f). Repeating the experiment with activation of recovery DANs without odors or with the unpaired odor failed to recover learned avoidance (Extended Data Fig. 2l). Thus, we conclude that recovery DANs can gate context-dependent memory recovery.

Together, these experiments demonstrate that KC-mediated retrieval of a silent memory, output from the multisensory LH and vesicle release from recovery DANs are essential for recovering forgotten memories. Furthermore, increased activation of recovery DANs can compensate for insufficient reminder conditions, facilitating memory recovery under suboptimal conditions.

### Memory recovery is mediated by a silent memory trace in specific MBONs

**Fig. 3:**
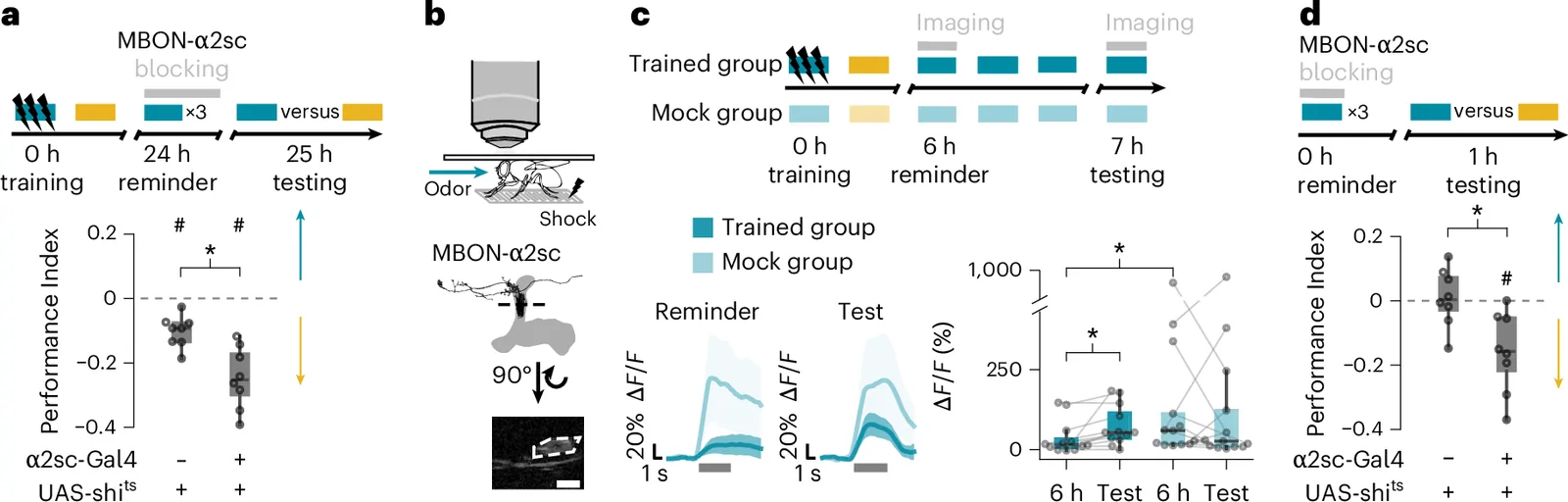
Memory recovery is mediated by a silent memory trace in MBON-α2sc. **a**, Top: protocol. Bottom: blocking MBON-α2sc during reminders enhances memory recovery. For each group *n* = 8. **b**, Top: illustration of the aversive training setup for head-fixed flies under 2-photon microscope. Bottom: MBON-α2sc imaging plane (top) and example frame (bottom; region of interest for quantification denoted with a white dashed line). Scale bar: 10 μm. **c**, Top: protocols for trained and mock groups. Bottom: trained flies show a reduced odor response to paired odor at the time of first reminder, but not at the time of test. Left: MBON-α2sc odor-evoked calcium responses at different time points. Mean and s.e.m. are respectively represented as a solid line and shadow; gray line indicates odor presentation window used for quantification. Right: plots show odor-evoked peak responses (Δ*F*/*F*) of individual flies represented as dots. Trained group: *n* = 12, mock group: *n* = 13. **d**, Top: protocol. Bottom: blocking MBON-α2sc in untrained flies during repeated odor exposures mimics memory recovery. For each group *n* = 8. Asterisk (*) indicates significant difference; hash (#) indicates difference from zero. All statistical tests and *P* values are reported in Supplementary Table 2. Data are presented as box and whisker plots showing the median, IQR and whiskers extending to the most extreme values within 1.5× IQR. Gal4-driver: MB080C for MBON-α2sc and empty-Gal4 for MBON-α2sc genetic control. Source data

**Extended Data Fig. 3:**
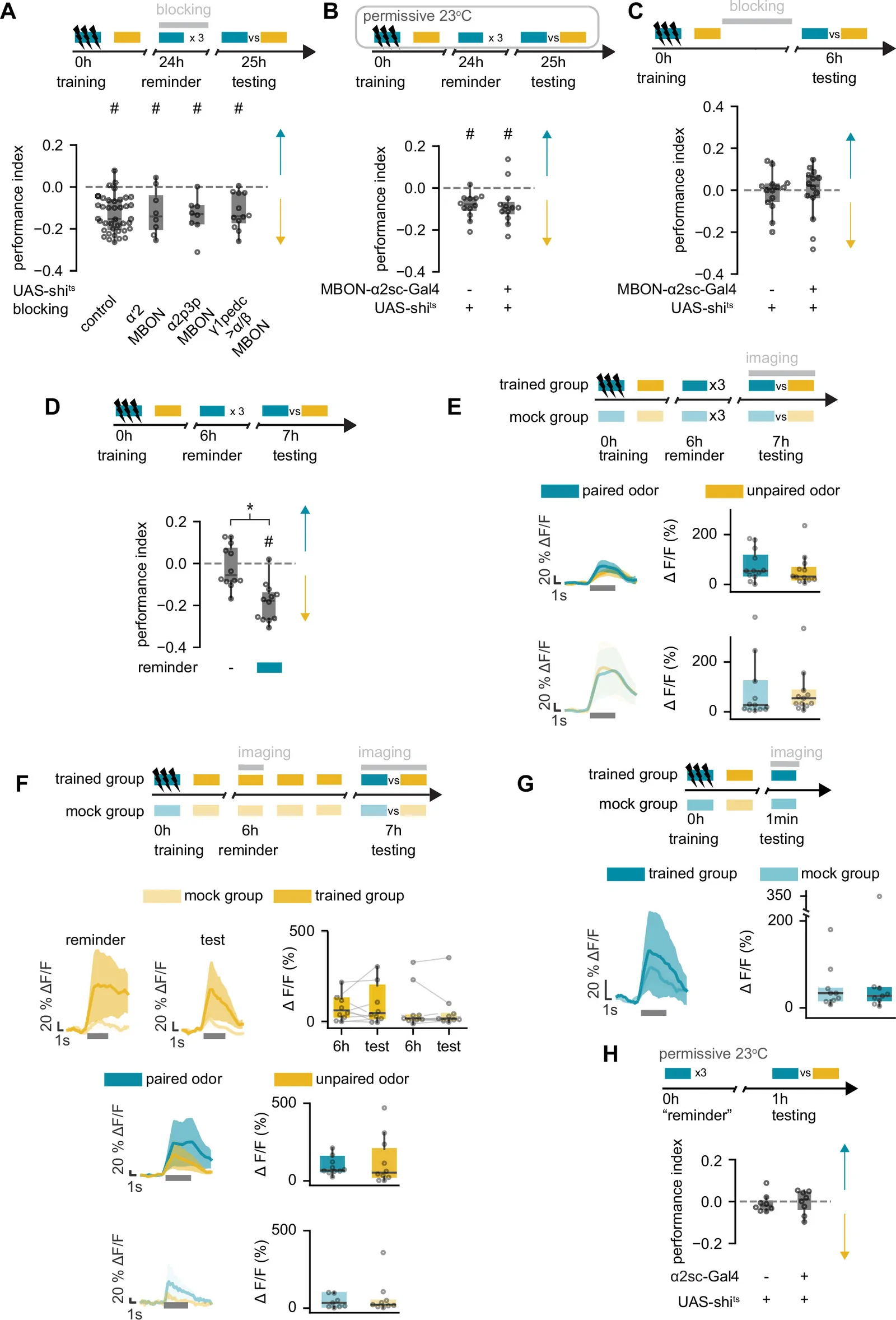
Memory recovery is mediated by a silent memory trace in MBON-α2sc. **a**) Top: protocol. Bottom: blocking MBON-α′2, MBON-α2p3p or MBON-γ1pedc > α/β during reminders does not impair memory recovery. Empty split-Gal4/Shi n = 40, MB018B/Shi n = 8, MB062B/Shi n = 8, MB085C/Shi n = 12. **b**) Top: protocol. Bottom: temperature control experiment from Fig. 3a; flies recover learned avoidance. For each group n = 12. **c**) Top: protocol. Bottom: Blocking MBON-α2sc neurons after training does not affect forgetting. For each group n = 14. **d**) Top: protocol. Bottom: repeated reminders recover forgotten memory at 7 h. For each group n = 12. **e**) Top: protocols for trained and mock groups. Bottom: No differences between paired (blue) and unpaired (yellow) odor presentations for both trained and mock-trained flies. On the left, average calcium activity for all odor responses represented as mean (solid lines) and SEM (shadow); gray line indicates odor presentation window used for quantification. On the right, odor-evoked peak responses (ΔF/F) of individual flies are represented as dots (right). Trained group n = 12, mock group n = 13. **f**) Top: unpaired odor reminder protocols for trained and mock groups. Middle: no training-induced nor unpaired odor reminder-induced plasticity is observed in MBON-α2sc. Bottom: no differences between paired and unpaired odor presentations. Trained group n = 10, mock group n = 9. **g**) Top: protocols for trained and mock groups. Bottom: no training-induced plasticity is observed when testing immediately after training in MBON-α2sc. For each group n = 9. **h**) Top: protocol. Bottom: temperature control experiment from Fig. 3d; flies show no avoidance. For each group n = 8. In panels A-D and H, asterisks indicate significant differences between groups and hash significant differences from zero. All statistical tests and *p values* are reported in Supplementary Table 2. Data are presented as box-and-whisker plots showing the median, interquartile range, and whiskers extending to the most extreme values within 1.5xIQR. Gal4-drivers: MB018B for MBON-α′2, MB062B for MBON-α2p3p, MB085C for MBON-γ1pedc > α/β, MB080C for MBON-α2sc and empty-Gal4 for genetic control in 3 A, 3B, 3 C and 3H. Source data

The MB is organized into compartments defined by the zonal innervation of DANs and MBONs. These compartments are highly interconnected via direct feedforward and recurrent feedback connections^44^. Thus, memory traces in the MB output system can modify the consequences of future experiences, including retrieval-driven memory re-evaluation^13^. Based on the connectivity of MB output circuits to recovery DANs (Extended Data Table 1) or their reported involvement in aversive memory retrieval^16,21,22,44^, we tested the role of targeted MB output circuits in the recovery process. Independently blocking MBON-α′2 or MBON-α2p3p, which connect highly to recovery DANs, or the approach-coding MBON-γ1ped>αβ during the reminder session did not impair memory recovery (Extended Data Fig. 3a). Surprisingly, blocking MBON-α2sc during reminders enhanced the recovery of learned avoidance (Fig. 3a). When experiments were conducted at permissive temperatures, memory recovery was unchanged (Extended Data Fig. 3b). In addition, blocking MBON-α2sc after learning did not prevent forgetting (Extended Data Fig. 3c). These findings indicate that MBONs-α2sc, a pair of behaviorally neutral MBONs, is involved in memory recovery.

MBON-α2sc has strong reciprocal connections with the recovery DANs (Extended Data Table 1). To investigate whether MBON-α2sc exhibits a memory trace that could support the reinstatement of odor-specific aversive memories, we adapted the recovery protocol to train head-fixed flies expressing the genetically encoded calcium reporter GCaMP7s^45^ under a 2-photon microscope (Fig. 3b). We adapted the recovery protocol to an earlier time point when aversive memory is behaviorally undetectable (Extended Data Fig. 3d) and provided the reminder session at 6 h. At the first reminder, compared to mock controls, trained flies showed a robust decrease in the peak response to the paired odor (Fig. 3c). Odor responses were again recorded 1 h following the reminder session, a time point when the avoidance behavior is recovered. However, at the time of this test, no differences in odor responses were observed (Fig. 3c and Extended Data Fig. 3e). Accordingly, within-animal comparisons showed that responses to the paired odor were lower during the first reminder trial than during the test in trained animals, while mock-treated animals exhibited comparable responses across the two sessions (Fig. 3c). Additional experiments revealed no differences in responses between trained and mock flies when the unpaired odor was presented during the reminders (Extended Data Fig. 3f). Consistent with previous studies^40^, imaging immediately after training showed no evidence of a memory trace in MBON-α2sc (Extended Data Fig. 3g). These findings demonstrate that a specific memory trace for the paired odor emerges over time in MBON-α2sc, correlating with the decay of learned behavior.

Together with the blocking experiments, these findings suggest that a silent memory trace, characterized by reduced output from MBON-α2sc, supports the recovery of forgotten aversive memories. To test the causal role of this silent memory trace, we artificially mimicked the reduced output from MBON-α2sc by blocking them during odor exposure in untrained flies. Remarkably, this imitation of the MBON-α2sc memory trace induced learned avoidance in a 1-h test (Fig. 3d). Conducting the same experiment at a permissive temperature did not result in avoidance behavior (Extended Data Fig. 3h). Previous work showed that the MBON-α2sc are neutral MBONs that do not mediate approach or avoidance behavior^17,22^. Together, these findings demonstrate that a silent memory trace in neutral MBON-α2sc emerges over time, mediating the recovery of forgotten memories. However, this silent memory trace appears to be absent after the reminder protocol, suggesting that additional plasticity elsewhere is required to guide behavior.

### Recovery reinstates aversive memory traces

**Fig. 4:**
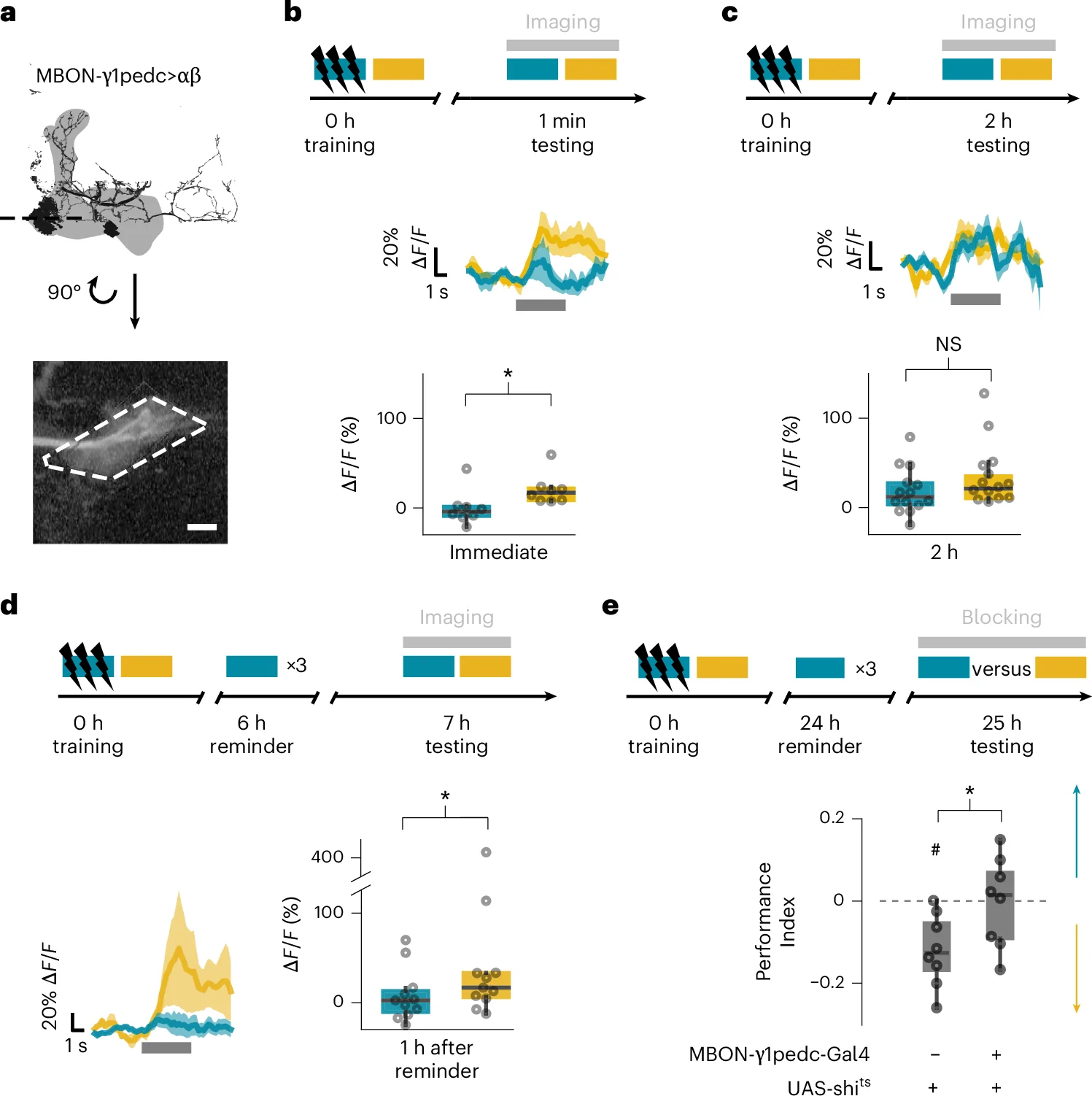
Recovery reinstates aversive memory trace in MBON-γ1ped>αβ. **a**, Top: MBON-γ1ped>αβ imaging plane. Bottom: example frame with region of interest for quantification denoted with a white dashed line. Scale bar: 10 μm. **b**, Top: protocol for imaging immediate training-induced plasticity. Bottom: training induces different responses to paired (blue) and unpaired (yellow). *n* = 8. **c**, Top: protocol for imaging training-induced plasticity at 2 h. Bottom: no differences are observed in the odor responses between paired (blue) and unpaired (yellow) odor. *n* = 14. **d**, Top: protocol for imaging reminder-induced plasticity. Bottom: reminders recover training-induced plasticity. *n* = 11. **e**, Top: protocol. Bottom: blocking MBON-γ1ped>αβ in trained flies during test abolishes retrieval of recovered memory. Data are presented as box and whisker plots showing the median, IQR and whiskers extending to the most extreme values within 1.5× IQR. For each group *n* = 8. In all imaging panels, MBON-γ1ped>αβ odor-evoked responses are represented as mean (solid) and s.e.m. (shadow); gray line indicates odor presentation window used for quantification. Box and whisker plots show odor-evoked mean responses (Δ*F*/*F*) of individual flies represented as dots. Gal4-drivers: MB112C (imaging experiments) or MB085C (behavioral experiments) for MBON-γ1ped>αβ, empty-Gal4 for MBON-γ1ped>αβ genetic control. Asterisk (*) denotes a significant difference; hash (#) indicates difference from zero. All statistical tests and *P* values are reported in Supplementary Table 2. Source data

**Extended Data Fig. 4:**
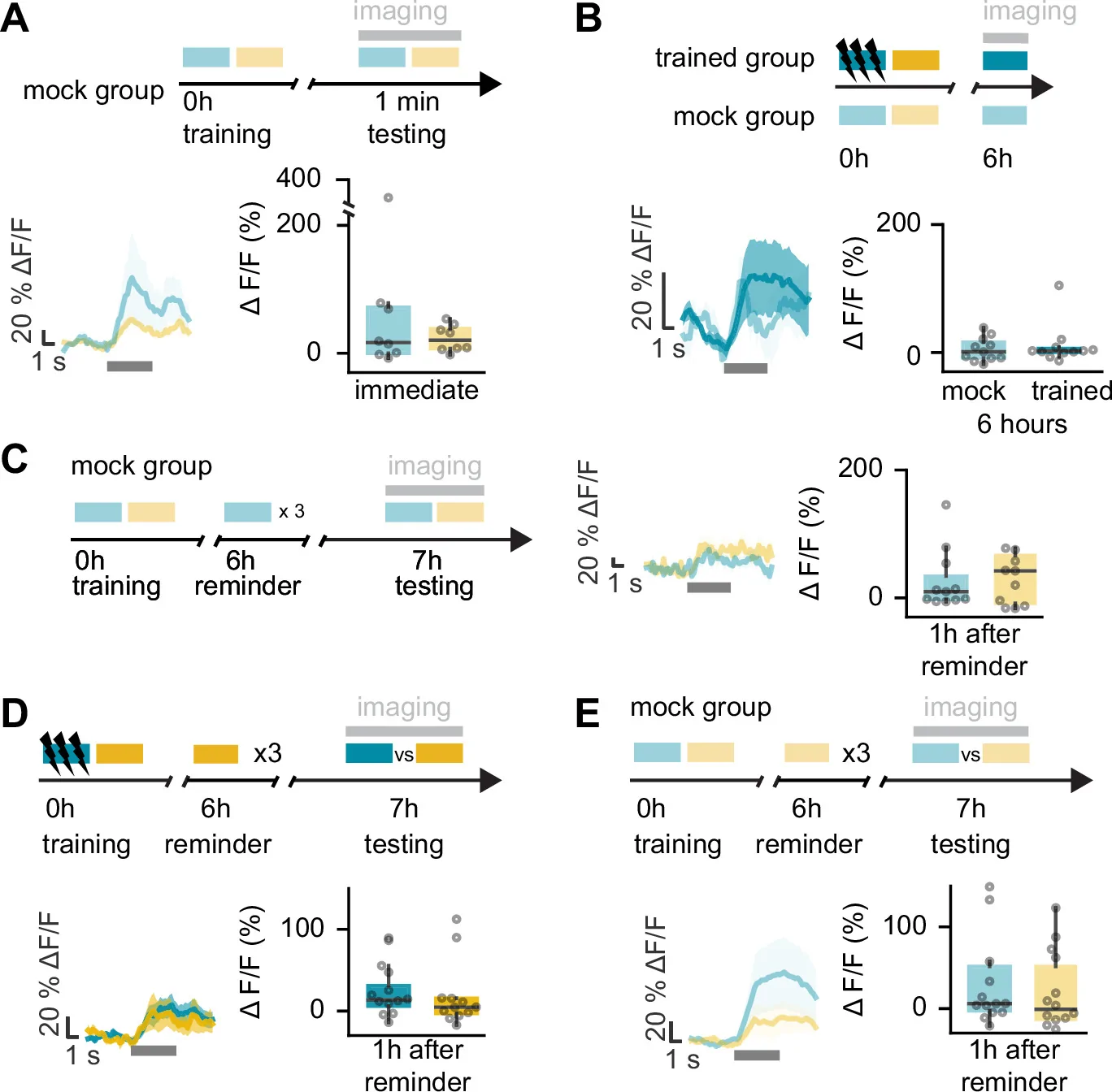
Recovery reinstalls aversive memory trace in MBON-γ1ped > αβ. **a**) Top: protocol. Bottom: mock-trained flies show no differences in odor-evoked responses. For each group n = 8. **b**) Top: protocols for imaging trained and mock groups. Bottom: no differences in paired odor responses between mock and trained animals at first reminder trial. For each group n = 11. **c**) Left: protocols for imaging mock group following reminder session. Right: mock-trained flies show no differences in odor-evoked responses. For each group n = 11. **d**) Top: unpaired odor reminder protocols for trained group. Bottom: unpaired odor reminder shows no differences between paired and unpaired odor responses. For each group n = 12. **e**) Top: unpaired odor reminder protocols for mock group. Bottom: unpaired odor reminder shows no differences between paired and unpaired odor responses. For each group n = 12. Gal4-drivers: MB112C for MBON-γ1ped > αβ. In all plots, mean and SEM are respectively represented as a solid line and shadow; gray line indicates odor presentation window used for quantification. Mean odor-evoked average responses (ΔF/F) of individual flies are represented as dots (right). All statistical tests and *p values* are reported in Supplementary Table 2. Source data

As behavioral expression decays, a silent memory trace emerges over time. Therefore, we next investigated whether other memory traces, critical for aversive behavior expression, exhibit a temporal dynamic that aligns with changes in memory retrievability. Previous studies have shown that aversive training leads to a depression of the approach-coding MBON-γ1ped>αβ and that this plasticity is critical for the expression of aversive memories^18,21^. We tested whether this active memory trace decays over time and can be recovered by reminders. Consistent with previous studies, our experiments showed that immediately after training, responses in MBON-γ1ped>αβ to the paired odor are reduced compared to the unpaired odor (Fig. 4a,b). In mock-trained flies, odor responses did not differ in the immediate test (Extended Data Fig. 4a). However, as previously shown^27,46^, when we tested the memory trace in MBON-γ1ped > αβ 2 h after training, it was undetectable, showing that this trace decays over the first hours after training (Fig. 4c). We next tested whether odor-specific memory traces are re-established by reminders. Testing responses at the first reminder to the paired odor in MBON-γ1pedc>αβ, 6 h after training, did not differ between mock-trained and trained flies (Extended Data Fig. 4b). However, at the test conducted after the reminders (7 h post-training), trained flies exhibited reduced responses to the paired odor compared to the unpaired odors (Fig. 4d). These differences were absent in mock-trained flies or when trained flies were re-exposed to the unpaired odor (Extended Data Fig. 4c–e). Thus, exposing trained flies to the reminder at 6 h post-training reinstates memory traces in approach-coding MBON-γ1pedc>αβ. Finally, we tested whether MBON-γ1pedc>αβ is involved in retrieval of the recovered aversive memory. Though blocking MBON-γ1pedc>αβ during reminders leaves recovery unchanged (Extended Data Fig. 3a), its synaptic output is required to retrieve the recovered memory (Fig. 4e).

Together, these findings indicate that plasticity in circuits guiding aversive memory retrieval decays over time while silent memory traces emerge. This silent state can be reversed, reinstating the retrievable memory trace when flies receive reminders that recover learned avoidance.

### Manipulation of the recovery protocol leads to false memory formation

**Fig. 5:**
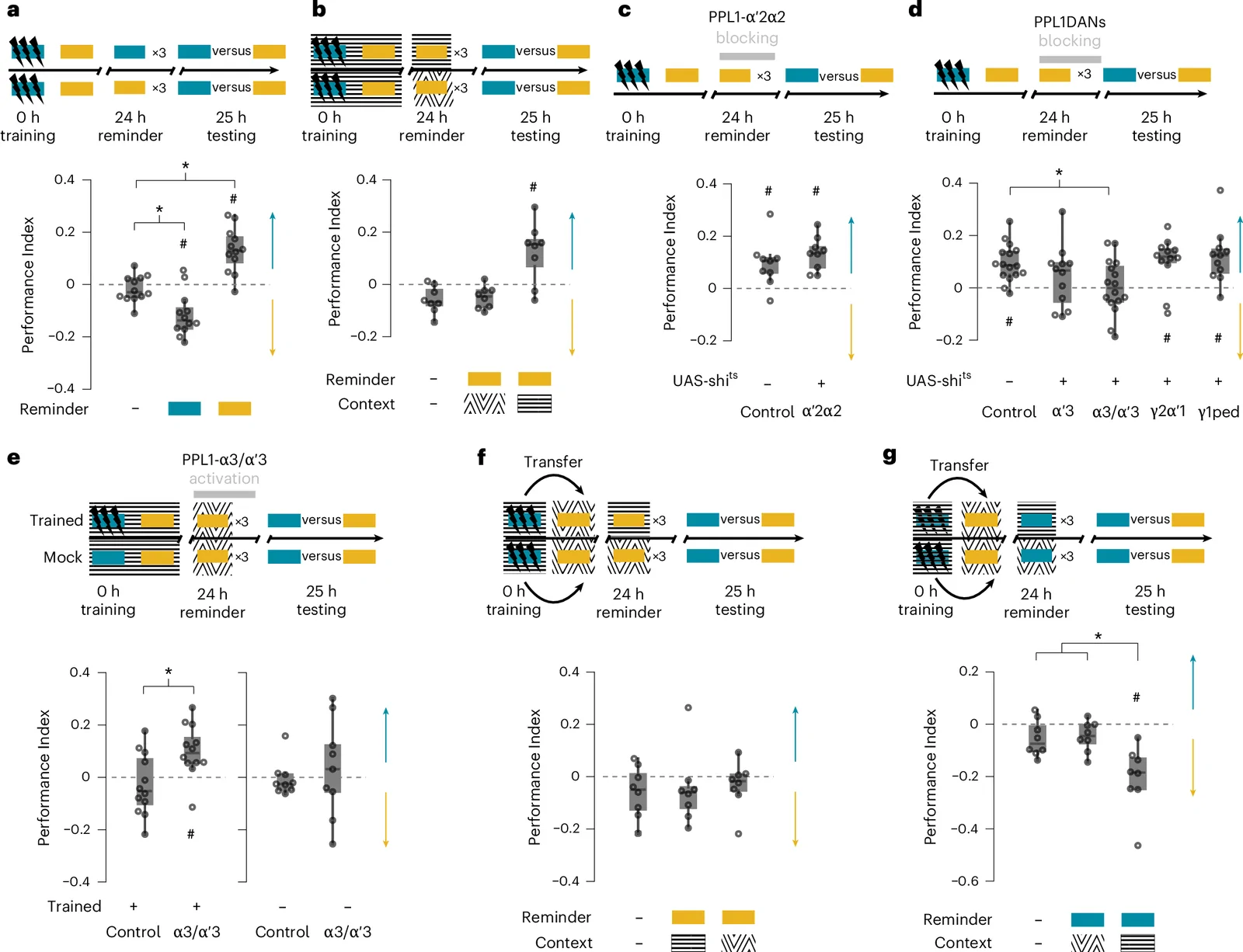
Manipulation of the reminder protocol leads to false memory formation. **a**, Top: protocols. Bottom: presenting the unpaired odor as a reminder leads to a false aversive memory. For each group *n* = 12. **b**, Top: protocols indicate same or different texture between training and reminders. Bottom: changing texture during reminders prevents false memory formation. For each group *n* = 8. **c**, Top: protocol. Bottom: blocking PPL1-α′2α2 recovery DANs during unpaired odor reminders does not prevent false memory formation. For each group *n* = 9. **d**, Top: protocol. Bottom: blocking PPL1-α3/α‘3 DANs during unpaired odor reminders prevents false memory formation. Empty split-Gal4/Shi: *n* = 16, MB304B/Shi: *n* = 12, MB630B/Shi: *n* = 12, MB296B/Shi: *n* = 12, MB320C/Shi: n = 11. **e**, Top: protocols. Bottom: activating PPL1-α3/α‘3 DANs during unpaired odor exposures in a different context supports false memory formation in trained flies but not in mock-trained flies. For both trained groups: *n* = 12, for both mock groups: *n* = 9. **f**, Top: protocols indicate the change of context (transfer) during training. Bottom: changing the context (texture) during training prevents false memory formation. For each group *n* = 8. **g**, Top: protocol indicates the transfer between chambers during the training. Bottom: changing the chambers but not the context does not prevent true memory recovery. For each group *n* = 8. Asterisk (*) denotes a significant difference between groups; hash (#) indicates difference from zero. All statistical tests and *P* values are reported in Supplementary Table 2. Data are presented as box and whisker plots showing the median, IQR and whiskers extending to the most extreme values within 1.5 x IQR. Gal4-driver: MB630B for PPL1-α3/α‘3, MB304B for PPL1-α‘3, MB320C for PPL1-γ1pedc, MB296B for PPL1-γ2α‘1, MB058B for PPL1-α‘2α2 and empty-Gal4 for PPL1 genetic control. Source data

**Extended Data Fig. 5:**
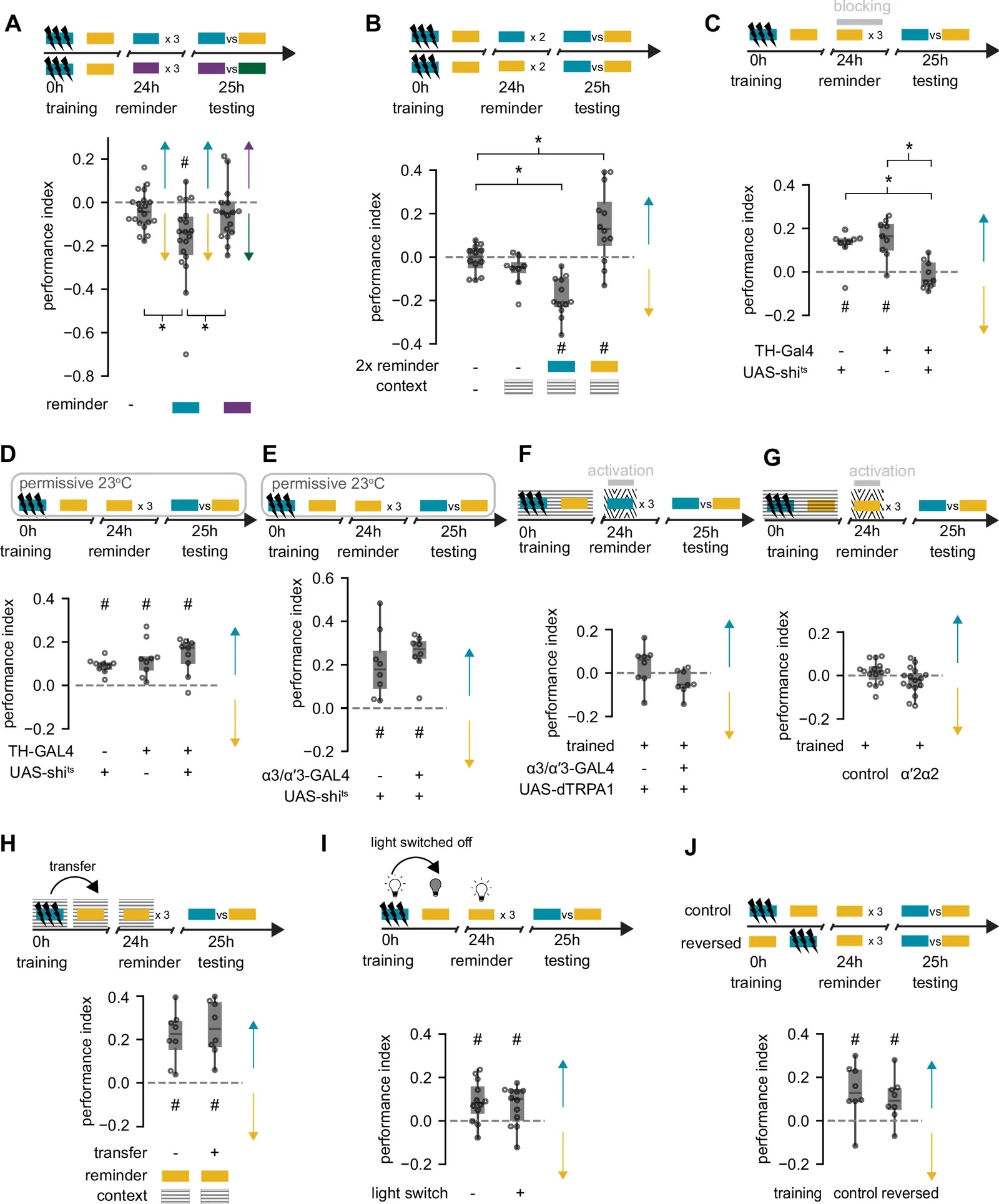
Manipulation of the recovery protocol can lead to false memory formation. **a**) Top: protocol indicates the use of different odors. Bottom: presenting a novel odor as a reminder fails to change behavior. No reminder control n = 20, CS+ as reminder n = 19, novel odor as reminder n = 17. **b**) Top: protocol. Bottom: two reminder trials with the unpaired odor leads to false memory formation. No reminder control n = 12, Only context as reminder n = 8, CS+ as reminder n = 12, CS- as reminder n = 12. **c**) Top: protocols. Bottom: blocking TH-Gal4 neurons during unpaired odor reminders prevents false memory formation. CS/Shi n = 8, TH-Gal4/CS n = 9, TH-Gal4/Shi n = 9. **d**) Top: protocols. Bottom: temperature control experiment from Extended Data Fig. 3c; unpaired odor reminder leads to false memory formation. For each group n = 9. **e**) Top: protocol. Bottom: temperature control experiment from 5D; unpaired odor reminders leads to false memory formation. For each group n = 8. **f**) Top: protocol indicating the activation of PPL1-α3/α‘3 neurons in a different context during paired odor presentation. Bottom: activating the PPL1-α3/α‘3 neurons during paired odor reminders fails to create aversive memory. For each group n = 8. **g**) Top: protocol. Bottom: activating PPL1-α′2α2 recovery DANs with the unpaired odor does not lead to false memory formation. Empty split-Gal4/Trp n = 15, MB058B/Trp n = 16. **h**) Top: protocol. Bottom: transferring flies to a different context (texture) during training does not affect false memory formation. For each group n = 8. **i**) Top: protocol. Bottom: changing the light conditions during training does not prevent false memory formation. For each group n = 12. **j**) Top: protocols. Bottom: reversed training sequence does not prevent false memory formation. For each group n = 8. In all panels, asterisks indicate significant differences between groups and hash significant differences from zero. All statistical tests and *p values* are reported in Supplementary Table 2. Data are presented as box-and-whisker plots showing the median, interquartile range, and whiskers extending to the most extreme values within 1.5xIQR. Gal4-driver: MB630B for PPL1-α3/α‘3, MB304B for PPL1-α‘3, MB320C for PPL1-γ1pedc, MB296B for PPL1-γ2α‘1, TH-Gal4 for TH neurons and empty-Gal4 for genetic control in panels **e-g**. Source data

We show that reminders can reinstate aversive memories from forgotten silent memories (Fig. 1 and Extended Data Fig. 1). Previous studies in humans have shown that suggestive reminders can influence memory reconstruction leading to a recovery that deviates from the ground truth^6^. Given that memory retrieval requires an interaction between external stimuli and stored memory traces^47^, we next tested whether a modified reminder protocol can lead to false memory formation in flies. Trained flies were exposed to reminders including either the paired, the unpaired or a new odor 24 h after training. An hour later, we tested the avoidance behavior to these odors. As observed in the previous experiments, three reminders with the paired odor successfully reinstated forgotten memories in both experiments (Fig. 5a and Extended Data Fig. 5a). In contrast, three presentations of a new odor in the training context did not elicit avoidance behavior during testing (Extended Data Fig. 5a). However, re-exposure to the unpaired odor as a reminder resulted in its avoidance during the test (Fig. 5a). These findings were further confirmed in an experiment involving two reminders (Extended Data Fig. 5b). Similar to ‘true’ memory recovery, presenting the unpaired odor in a different context failed to induce avoidance behavior during testing (Fig. 5b). These results indicate that re-exposure to the unpaired odor acted as a suggestive reminder and caused it to acquire a negative valence, a false memory.

We next investigated whether false memory generation is gated by DANs in a manner similar to ‘true’ memory recovery. Surprisingly, we found that blocking recovery DANs (PPL1-α‘2α2) during re-exposure to suggestive reminders did not affect false memory generation (Fig. 5c). However, blocking all punishment DANs using the same protocol prevented false memory generation (Extended Data Fig. 5c). When the experiments were repeated at permissive temperatures, false memories were unaffected (Extended Data Fig. 5d). Focusing on individual PPL1 DANs, we observed that false memory formation was abolished when the two PPL1-α3/α′3 were blocked during unpaired odor re-exposure. In contrast, blocking PPL1-γ1ped, or PPL1-γ2α′1 DANs had no effect. When only PPL1-α′3 DANs were blocked, false memory formation appeared to be partially reduced (Fig. 5d). Importantly, conducting these experiments at permissive temperatures leaves false memory intact (Extended Data Fig. 5e). These findings demonstrate that false memory generation depends on PPL1-α3/α′3 DANs, highlighting that recovery and false memory formation engage distinct dopaminergic pathways.

To further explore whether PPL1-α3/α′3 DANs gate false memory formation similarly to how recovery DANs gate the re-emergence of the true memory in insufficient reminder conditions, we conducted additional experiments. Trained flies received three presentations of the unpaired odor in different contexts (smooth chambers) while PPL1-α3/α′3 DANs were thermogenetically activated 24 h after training. As expected, under insufficient reminder conditions, genetic controls showed no evidence of false memory formation. However, activating PPL1-α3/α′3 DANs during unpaired odor re-exposure established avoidance behavior toward the unpaired odor in trained but not in mock-trained flies (Fig. 5e). These findings indicate that false memory formation is gated by activity in specific DANs, analogous to how recovery DANs support the re-emergence of ‘true’ memories.

To investigate the specificity of this gating, we tested whether activating PPL1-α3/α′3 DANs could support the recovery of ‘true’ memories or activating recovery DANs could support false memory formation. In two independent experiments, flies expressing dTRPA1 in either PPL1-α3/α′3 DANs or recovery DANs were trained on the first day. On the following day, flies were exposed to reminders in a different context. In one experiment PPL1-α3/α′3 DANs were activated during presentations of the paired odor (true memory reminder) (Extended Data Fig. 5f). In the other experiments, recovery DANs were activated during the re-exposure to the unpaired odor (suggestive reminder) (Extended Data Fig. 5g). In both experiments, the activation of the respective DANs failed to induce odor avoidance behavior. Together with the experiments that show that re-exposure to the unpaired odor failed to establish a memory trace in MBON-γ1ped>αβ (Extended Data Fig. 4d,e), these findings demonstrate that true memory recovery and false memory formation are separable processes, each gated by distinct pathways.

### Contextual experience during training defines the potential of false memory formation

Previous studies suggest that the plausibility of the suggestive information influences the potential to form a false memory^48^. In our experiments, we found that the ability of an odor to act as a suggestive reminder is context-dependent and appears to be shaped by experience during training. To investigate whether contextual changes during training impact false memory generation, flies were trained on the first day using a modified protocol. Here, the paired odor and electric shock were given in the training chamber, while the unpaired odor was presented in a chamber with a smooth surface. On the following day, the unpaired odor was presented as a suggestive reminder in either the training chamber or the smooth chamber, followed by a test 1 h later. Under this modified protocol, the unpaired odor failed to induce false memory formation (Fig. 5f). However, presenting the paired odor as a reminder after the same modified training protocol recovers the ‘true’ memory (Fig. 5g). Notably, these experiments required transferring flies from the training chambers to smooth chambers during training. We conducted additional experiments to test whether the transfer alone influenced false memory formation. Flies were subjected to the same handling but were transferred into chambers that matched the training context before receiving the unpaired odor. In this scenario, false memory formation remained intact (Extended Data Fig. 5h). In contrast to changes in texture, altering the light conditions during training did not significantly affect the capacity for false memory formation (Extended Data Fig. 5i). Furthermore, reversing the training sequence—where flies were first exposed to the unpaired odor in the training context and then to the paired odor with electric shocks—did not impair false memory formation (Extended Data Fig. 5j). Together, these findings demonstrate that suggestive reminders can lead to false memory formation. However, context-related experiences during training events critically influence how suggestive reminders mislead memory recovery.

## Discussion

**Extended Data Fig. 6:**
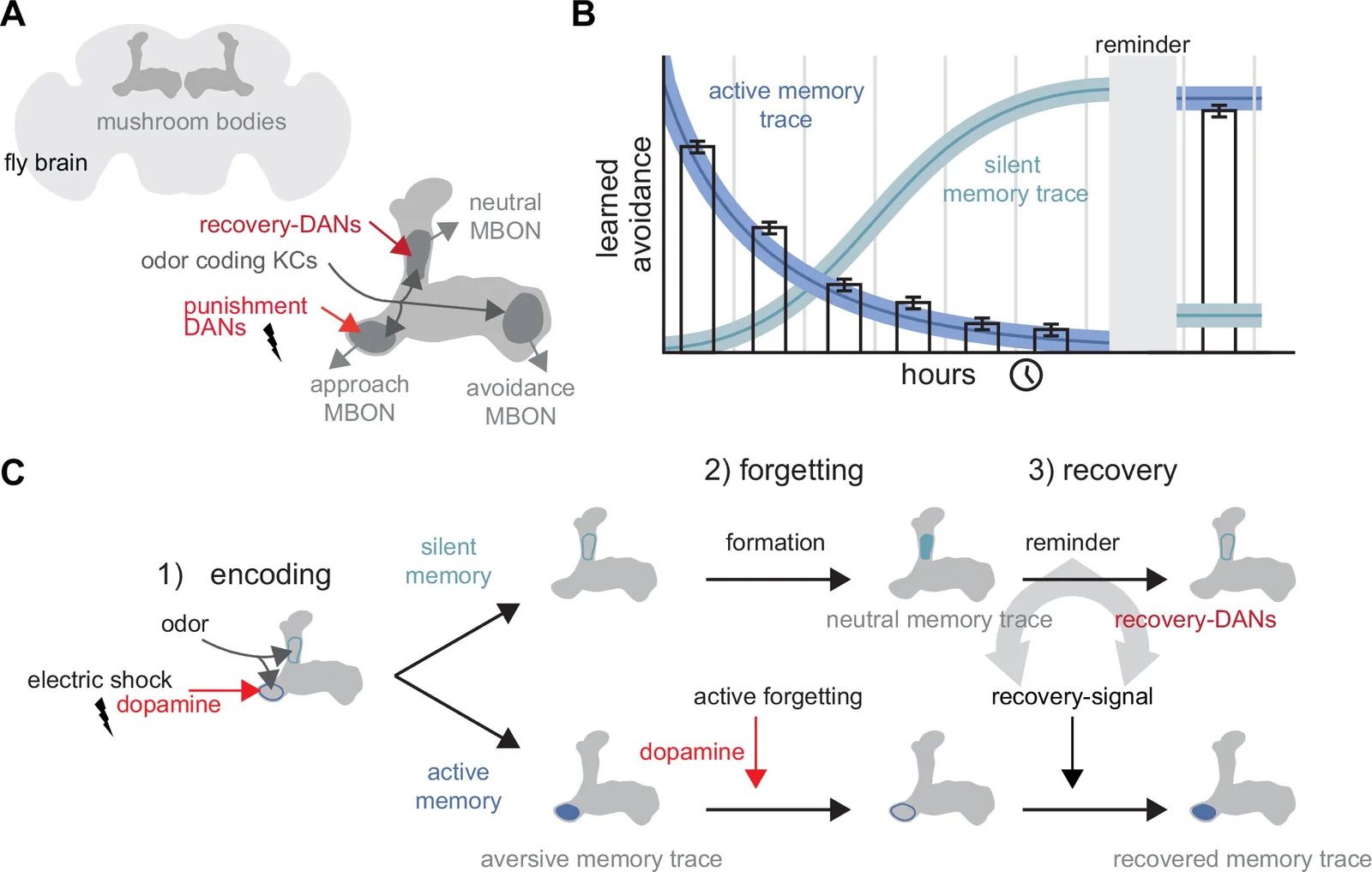
Working model for silent memory storage and reminder-driven recovery in the mushroom body. **a**) Schematic of the mushroom body (MB) circuit elements investigated. Odors are represented by activity in Kenyon cells (KCs), which provide input to MB output neurons (MBONs). During aversive learning, punishment-signaling dopaminergic neurons (DANs; red) convey electric-shock reinforcement and induce synaptic plasticity at KC → MBON connections. After training, an active aversive memory trace in approach-coding MBONs supports learned avoidance, whereas a persistent silent trace emerges in behaviorally neutral MBONs that do not directly drive odor-guided approach or avoidance. A distinct pair of PPL1-α′2α2 DANs (‘recovery DANs’) is selectively required during reminders to gate memory recovery. **b**) Conceptual time course of behavior and circuit state. Learned avoidance decays over hours after training in parallel with a reduction of the active memory trace, rendering the memory behaviorally inaccessible. In contrast, a silent memory trace emerges during the forgetting period. Presentation of an appropriate reminder (paired odor in the training context) triggers recovery, reinstating learned avoidance and the active trace. **c**) Stepwise model of encoding, forgetting, and recovery. 1) Encoding: Coincident odor-driven KC activity and punishment-related dopamine initiate plasticity, establishing both an early active aversive trace in approach-coding MBONs and a latent silent trace in a neutral MBON pathway. 2) Forgetting: Ongoing network activity promotes decay of the active trace (active forgetting), while the silent trace becomes prominent over time. 3) Recovery: Re-exposure to the training context together with the paired odor generates a recovery signal that reinstates an active, behaviorally-expressing aversive trace (‘recovered memory trace’). While the paired odor engages KC output and the silent-trace pathway, recovery DANs gate this process assumingly by the integration of contextual information. Nevertheless, the precise nature of the recovery signal remains to be determined.

In healthy conditions, forgetting is considered an adaptive process that allows the prioritization of reliable learned information. However, this prioritization comes at the cost of information loss^2^. To mitigate this loss, memories can be maintained in a silent yet recoverable state. At the circuit level, information loss appears to occur either through the decay of memory traces or by impaired access to them^1,49^. How these changes can be reconciled with the ability to recover forgotten memories has remained unclear. We find in *Drosophila* that forgotten memories can persist as silent memory traces in circuits that lack the capacity to guide behavior. From these silent memories, reminders can recover learned information by reinstating active memory traces in circuits that allow the expression of learned avoidance (Extended Data Fig. 6a–c). Thus, we conclude that switching between silent and active memory states prevents information loss and establishes a conditional memory system that allows the brain to flexibly prioritize memory retrieval based on recent experiences.

Consistent with previous studies in flies^27,46^, we found that during forgetting, memory traces in the approach-coding MBON-γ1ped>αβ decay over time. This decay is modulated by post-training experiences that recruit specific DANs and the activation of the small Rho GTPase Rac1^23–25,46,50^. However, rather than leading to permanent memory loss, this decay is accompanied by the emergence of a silent trace in behaviorally neutral MBON-α2sc. The delayed emergence of the silent trace is reminiscent of previous findings in *Drosophila* showing that consolidated long-term memory traces that drive behavior are initiated during training but only become evident over time^51–54^. Thus, we propose that, rather than memory being transferred between compartments during forgetting, both the active and the silent traces are initiated during training but differ in the temporal dynamics of when they are established. Given that the formation of long-term memories in the MB requires ongoing neuronal activity^55–58^, it is plausible that the post-training activity that promotes the decay of the active memory trace might simultaneously support the emergence of a silent trace. Dissecting whether these processes are mechanistically linked and how experience can modulate them will be key to understanding how remembering and forgetting are balanced.

From the silent state, we find that reminders can reinstate active memory traces in approach-coding MBONs, thereby recovering learned behavior. How can such a switch back to the active memory traces be established? The reminder-induced recovery is strictly contingent on re-exposure to both the training context and the paired odor. This specificity suggests that a coincidence of memory-related sensory inputs during the reminder elicits a recovery signal that reactivates an otherwise inaccessible memory. While the precise nature of this recovery signal remains unresolved, its odor specificity is most likely conveyed by KC output together with the associated silent trace within MBON-α2sc. In contrast, the contextual component likely arises from multisensory pathways, including LH circuits that interface with the MB output system and, in particular, with the silent memory pathway^28,39^. Central to the recovery process are the recovery DANs (PPL1-α‘2α2). These DANs do not support aversive learning and lack the ability to act as punishment-signaling neurons. Nonetheless, they are required for memory recovery, and their activation can compensate for insufficient contextual cues during the reminder. It is therefore plausible that they act as a central integrator that gates pathways in the α2 compartment to jointly generate the recovery signal. Consistent with the idea that memory recovery is not a simple recapitulation of initial acquisition, DANs that normally mediate punishment and can directly induce aversive learning are not required during recovery. This leads us to speculate that, although the activity-based MBON-γ1ped>αβ memory trace decays, longer-lasting molecular changes may persist in specific KCs and support its recovery independent of the classical teaching signal. Such a tagging of neurons and synapses can support memory allocation^59^ and would be in line with findings in mice and worms showing that forgetting leaves a molecular trace that favors memory recoverability^60,61^. Consistent with this idea, we find that memory recovery requires protein synthesis during and after training. In this framework, the reminder-induced recovery signal would reinstate accessibility by translating the persistent molecular tag into a retrievable trace, biasing the MB output system toward odor avoidance. In parallel to reinstating the active trace, we find that presenting the reminder appears to diminish the silent memory trace. Thus, it is worth considering whether the presence and absence of the active and silent memory traces are interlinked. Such competition between memory traces has been described in *Drosophila*^62^ and supports the view that forgetting and memory recovery arise from competition between engrams^49^.

Rodent studies show that forgetting reflects reduced access to memory engrams, but that increasing engram-cell excitability can restore memory expression^63–67^. Further, a recent study showed that forgotten memories formed in an object recognition task can be recovered by re-exposure to reminder cues and that this increased retrievability is accompanied by an enhanced activation of the original engram cells^68^. These findings indicate that, as in flies, engrams can persist in a latent or silent state that can be recovered by reminders. However, whether this silent state also involves a redistribution of engrams into circuits that do not drive behavior but facilitate later recovery, as we observe in *Drosophila*, remains to be investigated.

An important question raised by our results is why repeated presentation of the paired odor as a reminder leads to recovery rather than memory extinction (decrease of learned responses as a consequence of repeated unreinforced re-exposure). Previous work in *Drosophila* has shown that when an aversive memory is still retrievable, re-exposure to the paired odor recruits the active memory trace in MBON-γ1ped>αβ and engages a recurrent feedback pathway that drives extinction learning^69^. In the present study, however, reminders are delivered at a time when the original aversive memory is no longer behaviorally retrievable and the corresponding MBON-γ1ped>αβ trace is absent. Under these conditions the extinction circuit is unlikely to be engaged. Instead, reminder exposure appears to recruit a silent memory trace that reinstates the aversive association. Thus, the behavioral consequence of unreinforced stimulus exposure may depend on the circuit state of the memory, leading either to extinction or to recovery.

Considering memory recovery as part of an adaptive process^2,3^, a key question is why it sometimes permits the formation of memories that do not accurately reflect the contingencies during acquisition—resulting in false memories. We demonstrate that presenting the unpaired odor as a reminder leads to the formation of a false aversive memory. Notably, this capacity to form false memories is restricted to odors that were present in the training context, indicating that the potential for cues to distort memory recovery depends on their relation to the training event. In this regard, context defines the boundaries of what is considered part of the training. Because memory retrieval is susceptible to bias from external cues (ecphory)^47^, we argue that false memories arise from an inaccurate reconstruction of persistent silent memories. Indeed, our experiments demonstrate that the presence of either the paired or the unpaired odor during the reminders biases this reconstruction process. Furthermore, true memory recovery and false memory formation engage distinct DANs that project to different compartments. This separation of neuronal pathways implies that the respective memory traces are stored in distinct locations, arguably depending on whether the cues were predictive of the electric shock. Retaining all information from an aversive training episode as specific silent memories may increase flexibility to facilitate future learning and ultimately benefit animals’ survival. Accordingly, rather than being a flaw, we hypothesize that false memories emerge from a conditional memory system that, under other circumstances, promotes flexible adaptation based on experiences. Thus, it is intriguing to speculate that this adaptive process, permitting flexible reconstruction of memories, might represent a basic form of creativity.

## Methods

### Experimental model and subject details

#### Fly strains

Strains of *Drosophila melanogaster* were reared at 22 °C or 25 °C and 60%–65% humidity on standard cornmeal-agar food in 12:12 h light–dark cycle. Adult flies aged 3–9 days were used for all experiments. Canton-S flies were used as wild-type. Transgenes were expressed with fly lines produced by the Janelia FlyLight project^70^ or Vienna Drosophila Resource Center^71^. Males from Gal4-driver lines were crossed to virgins from the UAS. When split-Gal4 lines were used as drivers, an empty split-Gal4 line was used as genetic control. Flies used in the experiments shown in Extended Data Fig. 2k were kept and handled in the dark or under dim light conditions and transfered to food containing 125 mM all-trans Retinal 3 days before the experiment. All fly lines used are listed in the Key Resources Table (Supplementary Table 1).

### Method details

#### Behavioral experiments

Groups of approximately 100 flies were placed in a 25-ml vial containing standard food and a 20 × 60-mm piece of filter paper at least 8 h before behavioral experiments. Odors used in all experiments were 4-methylcyclohexanol, 3-octanol, isoamyl acetate or ethyl butyrate diluted in mineral oil. The odors were diluted to approximately 1:10^2^ or 1:10^3^ ratios for all experiments. Specifically, 7 μl 3-octanol, 12 μl–16 μl 4-methylcyclohexanol, 10 μl isoamyl acetate or 10 μl ethyl butyrate were diluted in 8 ml mineral oil. Except for thermogenetic experiments, all experiments were performed at 23 °C and 55–65% relative humidity. For *Shi*^*ts*^^*1*^ experiments^32^, the temperature was raised to the restrictive 30–33 °C at least 30 min before the targeted experimental phase (training, reminder or test). To activate targeted neurons through the dTRPA1 channels during the training or reminder session, we exposed flies to a high temperature of 32 °C only during the respective odor presentation. For optogenetic activation using CsChrimson, flies were exposed to red light stimulation (*λ* = 634 nm, 500 Hz, 25 µW per mm^2^).

Aversive olfactory conditioning in the T-maze was conducted as previously described^14,21^. However, different materials were used for making the shock chambers. Instead of copper-wires, the grid consists of silver conductive epoxy adhesive. Further, DS2A Isolated Voltage Stimulator (Digitimer Ltd) was used to deliver electric shocks. Groups of flies were transferred into shock chambers and exposed to a paired odor for 1 min, accompanied by 12 electric shocks (90V) at 5-s intervals. This was followed by 45 s of clean air and the presentation of a second odor (unpaired odor) for 1 min without shock. Unless stated otherwise, the presentation of both odors and clean air was done in the shock chambers. In all experiments odors used as paired or unpaired odor alternated, and each data point represents the average performance scores from two reciprocally trained groups. Memory was subsequently assessed by testing flies for their odor preference between the unpaired and the paired odor in a T-maze for 1 min. The Performance Index was calculated as the difference between the number of flies in the paired odor arm and the number in the unpaired arm, divided by the total number of flies^14^. During each reminder trial, flies were transferred to the respective chambers and either the paired, the unpaired or a new odor (isoamyl acetate or ethyl butyrate were used as new odors) was presented for 1 min. In multiple-trial experiments, an intertrial interval of 15 min (start of the preceding trial to the start of the next trial) was used. The context was changed by using smooth tubes that lack grids (to alter texture), by shielding the chambers from room light, or by raising the ambient temperature. In between treatments, flies were kept in 25-ml food vials.

#### Calcium imaging

For imaging experiments, 3–8-day-old female and male flies were briefly anesthetized on ice for 10 s and mounted in a custom-made 3D-printed platform. The proboscis was restrained to minimize movements during recordings. The head capsule was immersed and opened in buffer solution at room temperature (5 mM TES, 103 mM NaCl, 3 mM KCl, 1.5 mM CaCl_2_, 4 mM MgCl_2_, 26 mM NaHCO_3_, 1 mM NaH_2_PO_4_, 8 mM Trehalose, 10 mM glucose, pH 7). Membranes and trachea above the recording site were surgically removed. Head-fixed flies were placed under a modified Thorlabs Bergamo II two-photon microscope. A constant air stream at 0.9 ml min^−1^ was directly applied to the antenna of the fly using an olfactometer (220 A Aurora Scientific) and a custom-made electric shock grid was positioned below the fly contacting its legs. For all flies, GCaMP7s fluorescence signal was measured in one arbitrarily chosen brain hemisphere. Fluorescence was excited using a Ti-Sapphire laser centered at 920 nm. Laser power under the objective was set between 10 to 25 mW, depending on the sample. Images of 750 × 400 pixels (approximately 300 × 300 µm) were acquired at a frequency of 60 Hz using custom software^72^. Stimuli delivery and synchronized imaging acquisition were controlled using Stytra^73^. Flies were trained under the 2-photon microscope using either 4-methylcyclohexanol or 3-octanol paired with 90-V electric shock to the legs for 1 min. Following 45 s of air, the reciprocal odor was presented without the electric shock. Mock-trained flies were exposed to the same protocol but not presented with electric shock punishment during the whole experiment. Following the training session, head-fixed flies together with the recording platforms were moved to a different environment and left undisturbed for 6 h. After the 6 h period, flies were moved back to the same microscope training setting and exposed to three trials lasting 1 min each of either the paired odor or the unpaired odor with a 15-min intertrial interval. During the 15-min intertrial interval, head-fixed flies were removed from the microscope. Repeated removal and repositioning of head-fixed flies during the experimental protocol may introduce variability in odor-evoked responses across trials and experiments.

Two-photon fluorescence images were manually segmented using the Python Libraries EasyROI and OpenCV. When selecting the region of interest, a background subtraction was performed. Movement correction was performed, after XY binning, using Python Library CaImAn^74^. The fluorescence over the defined region of interest was averaged at each frame to yield one fluorescence trace, *F*(*t*), over time, *t*. Traces were then binned in time, reaching a final frequency of 4 Hz. All subsequent analyses were performed over the binned data using custom-written Python notebooks. Baseline fluorescence (*F*) corresponds to the average fluorescence signal during a 5-s window before each odor delivery. The baseline was then used to compute the relative change in fluorescence during the experiment (Δ*F*(*t*)/*F* = (*F*(*t*)*F*)/*F*). Fluorescence responses were quantified as the peak of the normalized activity within 5 s after odor onset for MBON-α2sc. For MBON-γ1pedc, the average of the normalized activity during the 5-s odor presentation.

#### Connectivity analysis

Connectivity analysis was performed using the hemibrain:v1.2.1 dataset^75^ via the NeuPrint user interface. Number of synapses between PPL1-*α*′2 *α*2 (PPL105) and MBONs were obtained through a custom query in NeuPrint. These data were used to construct Extended Data Table 1, which summarizes synapse numbers between PPL1-*α*′2 *α*2 (PPL105) and MBONs. Due to the limitations in the hemibrain dataset, only neurons from the right hemisphere were analyzed.

#### Quantification and statistical analysis

Statistical analyses were performed in Python Jupyter notebook. To identify significant approach or avoidance behavior in the final retrieval test, the performance of each group was tested with a one-sample *t*-test against zero. Data with multiple control groups (for example, two genetic controls) were analyzed with ANOVA followed by Tukey’s post hoc test. Data with one control group and one experimental group were analyzed with an unpaired *t*-test. No statistical methods were used to determine the sample size. All statistical tests, *n* numbers and *P* values can be found in Supplementary Table 2.

#### Statistics and reproducibility

Sample sizes were chosen in accordance with standard practices in the field. The sample size for each experiment is reported in the corresponding figure legends and in Supplementary Table 2. Experiments were independently replicated across multiple days to ensure reproducibility before data were considered valid. Multiple genetic lines of *Drosophila melanogaster* were used in this study, and a complete list of all lines is provided in Supplementary Table 1. Animals of both sexes were included without sex-based selection bias. No statistical method was used to predetermine sample size. No data were excluded from the analyses unless predefined quality-control criteria were not met (for example, behavioral trials with fewer than 20 flies were excluded before analysis). The experiments were not randomized. The investigators were not blinded to allocation during experiments and outcome assessment.

### Reporting summary

Further information on research design is available in the Nature Portfolio Reporting Summary linked to this article.

## Online content

Any methods, additional references, Nature Portfolio reporting summaries, source data, extended data, supplementary information, acknowledgements, peer review information; details of author contributions and competing interests; and statements of data and code availability are available at https://doi.org/10.1038/s41593-026-02381-2.

## Supplementary information


Supplementary InformationSupplementary Tables 1 and 2.
Reporting Summary


## Source data


Source Data Fig. 1Performance Index.
Source Data Fig. 2Performance Index.
Source Data Fig. 3Performance Index for behavioral data, raw activity in the ROI for imaging data.
Source Data Fig. 4Performance Index for behavioral data, raw activity in the ROI for imaging data.
Source Data Fig. 5Performance Index.
Source Data Extended Data Fig. 1Performance Index.
Source Data Extended Data Fig. 2Performance Index.
Source Data Extended Data Fig. 3Performance Index for behavioral data, raw activity in the ROI for imaging data.
Source Data Extended Data Fig. 4Performance Index for behavioral data, raw activity in the ROI for imaging data.
Source Data Extended Data Fig. 5Performance Index.


## Figures and Tables

**Extended Data Table 1:**
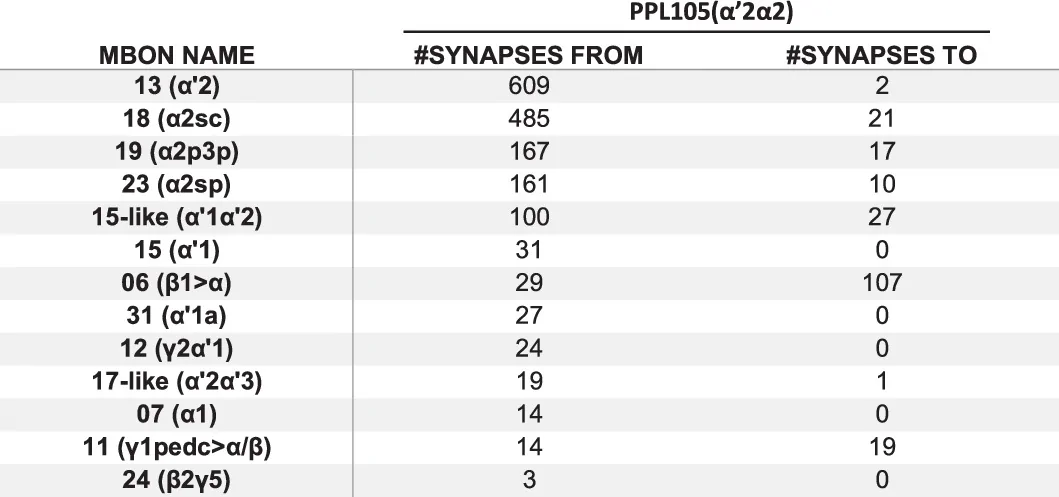
Synaptic connections between PPL105 and MBONs

## Data Availability

The experimental data that support the findings of this study are available via Zenodo at https://doi.org/10.5281/zenodo.20055473 (ref. ^76^). Source data are provided with this paper.

## Code availability

The complete analysis code is publicly available via Zenodo at https://doi.org/10.5281/zenodo.20055473 (ref. ^76^).
