# Supplementary material for: Creating true and false memories from forgotten information in Drosophila
Source: Nat Neurosci. 2026 Jul 30;29(9):2204–13. doi: 10.1038/s41593-026-02381-2 (PMC13533848; doi:10.1038/s41593-026-02381-2)
Supplement: Supplementary file 1 [file 41593_2026_2381_MOESM1_ESM.pdf]

nature neuroscience

Supplementary information https://doi.org/10.1038/s41593-026-02381-2

Creating true and false memories from forgotten information in *Drosophila*

In the format provided by the authors and unedited

Key Resources Table

REAGENT or RESOURCE	SOURCE	IDENTIFIER
**Chemicals, Peptides, and Recombinant Proteins**		
Active dry yeast	Sigma-Aldrich	Cat#789093
Mineral Oil	Sigma-Aldrich	Cat#M5904
4-methylcyclohexanol (98%)	Sigma-Aldrich	Cat#218405
3-octanol (99%)	Sigma-Aldrich	Cat#153095
Ethyl butyrate	Sigma-Aldrich	Cat#E15701
TES	Sigma-Aldrich	Cat#T1375
NaCl	Sigma-Aldrich	Cat#S7653
KCl	Sigma-Aldrich	Cat#P9333
NaHCO3	Sigma-Aldrich	Cat#S6297
NaH2PO4	Sigma-Aldrich	Cat#S8282
CaCl2	Sigma-Aldrich	Cat#21115
MgCl2	Sigma-Aldrich	Cat#M1028
Trehalose	Sigma-Aldrich	Cat#T9531
Glucose	Sigma-Aldrich	Cat#G7528
Silver conductive epoxy adhesive	MG-Chemicals	Cat#8331D
Isopentyl acetate (98%)	Sigma-Aldrich	Cat#112674
**Experimental Models: Organisms/Strains**		
Drosophila: Canton-S	Bloomington *Drosophila* Stock Center	RRID: BDSC_64349
Drosophila: R13F02-Gal4	Bloomington *Drosophila* Stock Center	RRID: BDSC_48571
Drosophila: R58E02-Gal4	Bloomington *Drosophila* Stock Center	RRID: BDSC_41347
Drosophila: TH-Gal4	Bloomington *Drosophila* Stock Center	RRID: BDSC_51982
Drosophila: MB058B-Gal4	Bloomington *Drosophila* Stock Center	RRID: BDSC_68278
Drosophila: MB320C-Gal4	Bloomington *Drosophila* Stock Center	RRID: BDSC_68253
Drosophila: MB630B-Gal4	Bloomington *Drosophila* Stock Center	RRID: BDSC_68334
Drosophila: MB296B-Gal4	Bloomington *Drosophila* Stock Center	RRID: BDSC_68308
Drosophila: MB018B-Gal4	Bloomington *Drosophila* Stock Center	RRID: BDSC_68296
Drosophila: MB062B-Gal4	Bloomington *Drosophila* Stock Center	RRID: BDSC_68280
Drosophila: MB549C-Gal4	Bloomington *Drosophila* Stock Center	RRID: BDSC_68373
Drosophila: MB085C-Gal4	Bloomington *Drosophila* Stock Center	RRID: BDSC_68288

Drosophila: MB077B-Gal4	Bloomington *Drosophila* Stock Center	RRID: BDSC_68283
Drosophila: MB080C-Gal4	Bloomington *Drosophila* Stock Center	RRID: BDSC_68285
Drosophila: MB310C-Gal4	Bloomington *Drosophila* Stock Center	RRID: BDSC_68313
Drosophila: LH989-Gal4	Bloomington *Drosophila* Stock Center	RRID: BDSC_86697
Drosophila: LH2193-Gal4	Bloomington *Drosophila* Stock Center	RRID: BDSC_86634
Drosophila: LH291-Gal4	Dolan et al., 2019[1]	N/A
Drosophila: LH1983-Gal4	Bloomington *Drosophila* Stock Center	RRID: BDSC_86644
Drosophila: LH1293-Gal4	Bloomington *Drosophila* Stock Center	RRID: BDSC_86672
Drosophila: LH1538-Gal4	Dolan et al., 2019[1]	N/A
Drosophila: NP1004-Gal4	KYOTO Stock Center (DGRC)	RRID: DGRC_112440
Drosophila: Empty split-Gal4	Bloomington *Drosophila* Stock Center;	RRID: BDSC_79603
Drosophila: Empty Gal4	Bloomington *Drosophila* Stock Center;	RRID: BDSC_68384
Drosophila: w1118	Bloomington *Drosophila* Stock Center;	RRID: BDSC_3605
Drosophila: UAS-dTrpA1	Bloomington *Drosophila* Stock Center	RRID: BDSC_26263
Drosophila: UAS-csChrimson	Bloomington *Drosophila* Stock Center;	RRID: BDSC_55135
Drosophila: UAS-RICIN	Bloomington *Drosophila* Stock Center;	RRID: BDSC_38623
Drosophila: UAS-Shi^{ts1}	Kitamoto 2001[1]	N/A
Drosophila: UAS-Gcamp7s	Bloomington *Drosophila* Stock Center	RRID: BDSC_79032
**Software and Algorithms**		
Adobe Illustrator CC	Adobe Systems, San Jose, CA	https://www.adobe.com/uk/products/illustrator.html
Iris 2-photon scanning	Keller Lab, FMI	https://sourceforge.net/projects/iris-scanning/
Stytra	Portugues Lab, MPI for neurobiology	https://portugueslab.com/stytra/
CalmAn calcium analysis	Flatiron Institute, USA	https://github.com/flatironinstitute/CaImAn
VisiView	Visitron Systems GmbH, Germany	https://www.visitron.de/products/visiviewr-software.html
Python	Python Software Foundation	https://www.python.org/
Jupyter Notebook	Jupyter Book Community[2]	https://jupyterbook.org/
NeuPrint	Janelia Research Campus[3]	https://neuprint.janelia.org/

Supplementary Table 2: key resources table

Contact for Reagent and Resource Sharing

Further information and requests for resources and reagents should be directed to and will be fulfilled by the Lead Contact, Johannes Felsenberg (johannes.felsenberg@fmi.ch)

References

References

1. Kitamoto, T. (2001). Conditional modification of behavior in Drosophila by targeted expression of a temperature-sensitive shibire allele in defined neurons. J Neurobiol *47*, 81-92. 10.1002/neu.1018.
2. Kluyver, T., Ragan-Kelley, B., Pérez, F., Granger, B.E., Bussonnier, M., Frederic, J., Kelley, K., Hamrick, J.B., Grout, J., and Corlay, S. (2016). Jupyter Notebooks-a publishing format for reproducible computational workflows.
3. Plaza, S.M., Clements, J., Dolafi, T., Umayam, L., Neubarth, N.N., Scheffer, L.K., and Berg, S. (2022). neuPrint: An open access tool for EM connectomics. Frontiers in Neuroinformatics *16*. 10.3389/fninf.2022.896292.

Experiment/ Figure	Group/Treatment	N	Normally distributed	statistical test	P value
Figure 1B	a) immediate	8	yes	one-sample t-test against zero	a) 0.0002<br>b) 0.0076<br>c) 0.0942
	b) 1.5h	8	yes		
	c) 24h	8	yes		
Figure 1C	a) no reminder	11	yes	one-sample t-test against zero; unpaired t-test	one-sample t-test:<br>a) 0.1607<br>b) 0.3742<br>c) 0.0023<br>d) 0.0005<br>e) 0.0026<br>f) 0.5437<br><br>unpaired t-test:<br>a) v b):0.9021<br>a) v c): 0.0056<br>a) v d): 0.0002<br><br>e) v f) 0.0117
	b) 1x reminder	9	yes		
	c) 2x reminders	9	yes		
	d) 3x reminders	9	yes		
	e) normal training with 3x reminders	9	yes		
	f) mock group with 3x reminders	9	yes		
Figure 1D	a) no reminder control	16	yes	one-sample t-test against zero; ANOVA+Tukey;	one-sample t-test:<br>a) 0.6227<br>b) 0.0003<br>c) 0.4519<br>ANOVA+Tukey:<br>a) v. b) 0.0053<br>a) v. c) 0.8560<br>b) v. c) 0.0219
	b) reminders in same texture	16	yes		
	c) reminders in the different texture	16	yes		
Figure 1E	a) no reminder control	12	yes	one-sample t-test against zero; ANOVA+Tukey;	one-sample t-test:<br>a) 0.9477<br>b) 0.0093<br>c) 0.9575<br><br>ANOVA+Tukey:<br>a) v. b) 0.0092<br>a) v. c) 0.9965<br>b) v. c) 0.0150
	b) light + reminders	8	yes		
	c) darkness + reminders	8	yes		
Figure 1F	a) 23 deg + no reminders	12	yes	one-sample t-test against zero; unpaired t-test	one-sample t-test:<br>a) 0.2562<br>b) <0.0001<br>c) 0.0337<br>d) 0.0004<br><br>unpaired t-test:<br>a) v. b) 0.0002<br>c) v. d) 0.0031
	b) 23 deg + reminders	12	yes		
	c) 32 deg + no reminders	12	no		
	d) 32 deg + reminders	12	yes		
Figure 2B	a) CS/Shi	11	yes		

	b) R13F02/CS	11	yes	one-sample t-test against zero; ANOVA+Tukey;	one-sample t-test:<br>a) <0.0001<br>b) 0.0004<br>c) 0.0004<br><br>ANOVA+Tukey<br>ANOVA p=0.3675<br>a) v. b) 0.9761<br>a) v. c) 0.5047<br>b) v. c) 0.3859
	c) R13F02/Shi	11	yes		
Figure 2C	a) CS/Shi	13	yes	one-sample t-test against zero; ANOVA+Tukey;	one-sample t-test:<br>a) 0.0013<br>b) < 0.0001<br>c) 0.4617<br><br>ANOVA: 0.0003<br>a) v b) 0.2451<br>a) v c) 0.0335<br>b) v c) 0.0002
	b) R13F02/CS	15	yes		
	c) R13F02/Shi	15	yes		
Figure 2D	a) Empty split-gal4/Shi	11	yes	one-sample t-test against zero; unpaired t-test	one-sample t-test:<br>a) 0.0001<br>b) <0.0001<br><br>unpaired t-test:<br>a) v. b) 0.3442
	b) MB058B/Shi	11	yes		
Figure 2E	a) Empty split-gal4/Shi	8	yes	one-sample t-test against zero; unpaired t-test	one-sample t-test:<br>a) <0.0001<br>b) 0.338177<br><br>unpaired t-test:<br>a) v. b) <0.0001
	b) MB058B/Shi	8	yes		
Figure 2F	a) Empty split-gal4/Trp + training	12	yes	one-sample t-test against zero; ANOVA+Tukey;	one-sample t-test:<br>a) 0.0144<br>b) 0.4340<br>c) 0.0091<br>ANOVA+Tukey:<br>ANOVA p=0.0013<br>a) v. b) 0.9437<br>a) v. c) 0.0017<br>b) v. c) 0.0096
	b) MB058B/Trp + mock training	8	yes		
	c) MB058B/Trp + training	10	yes		
Figure 3A	a) Empty split-gal4/Shi	8	yes	one-sample t-test against zero; unpaired t-test	one-sample t-test:<br>a) 0.0006<br>b) 0.0001<br>unpaired t-test:<br>a) v b) 0.0020
	b) MB080C/Shi	8	yes		
Figure 3C	a1) reminder 1 trained	12	no		Kruskal Wallis test

	a2) reminder 1 mock	13	no	Kruskal Wallis test;<br>Wilcoxon test	a1) v. a2) 0.0387<br><br>Wilcoxon test<br>a1) v. a3) 0.0015<br>a2) v. a4) 0.8394
	a3) test CS+ trained	12	yes		
	a4) test CS- mock	13	no		
Figure 3D	a) Empty split-gal4/Shi	8	yes	one-sample t-test against zero; unpaired t-test	one-sample t-test:<br>a) 0.7813<br>b) 0.0091<br>unpaired t-test:<br>a) v b) 0.0082
	b) MB080C/Shi	8	yes		
Figure 4B	a) CS+	8	no	Wilcoxon test	Wilcoxon test<br>a) v. b) 0.0078
	b) CS-	8	no		
Figure 4C	a) CS+	14	Yes	Wilcoxon test	Wilcoxon test<br>a) v. b) 0.2676
	b) CS-	14	No		
Figure 4D	a) CS+	11	yes	Wilcoxon test	Wilcoxon test<br>a) v. b) 0.0322
	b) CS-	11	no		
Figure 4E	a) Empty-split gal4/Shi	8	yes	one-sample t-test against zero; unpaired t-test	one-sample t-test:<br>a) 0.0064<br>b) 0.9502<br><br>Unpaired t-test:<br>a) v b) 0.0328
	b) MB085B/Shi	8	yes		
Figure 5A	a) no reminder control	12	yes	one-sample t-test against zero;<br>ANOVA+Tukey	one-sample t-test:<br>a) 0.2742<br>b) 0.0007<br>c) 0.0003<br><br>ANOVA+Tukey:<br>ANOVA: p<0.0001<br>a) v. b) 0.0095<br>a) v. c) 0.0001<br>b) v. c) <0.0001
	b) CS+ as reminders	12	yes		
	c) CS- as reminders	12	yes		
Figure 5B	a) no reminder control	8	yes	one-sample t-test against zero;<br>ANOVA+Tukey;	one-sample t-test:<br>a) 0.0159<br>b) 0.0129<br>c) 0.0210<br><br>ANOVA+Tukey:<br>ANOVA p=0.0002<br>a) v. b) 0.9771<br>a) v. c) 0.0004<br>b) v. c) 0.0006
	b) react in plastic chamber	8	yes		
	c) react in the shock chamber	8	yes		

Figure 5C	a) empty split-gal4/Shi	9	yes	one-sample t-test against zero; unpaired t-test	one-sample t-test:<br>a) 0.0134<br>b) 0.0003
	b) MB058B/Shi	9	yes		
Figure 5D	a) Empty split-gal4/Shi	16	yes	one sample t-test against zero;<br>unpaired t-test but only with control data points that were done in parallel	one-sample t-test:<br>a) <0.0001<br>b) 0.2447<br>c) 0.8701<br>d) 0.0034<br>e) 0.0034<br><br>unpaired t-test:<br>a) v. b) 0.1413<br>a) v. c) 0.0072<br>a) v. d) 0.8456<br>a) v. e) 0.4690
	b) MB304B/Shi	12	yes		
	c) MB630B/Shi	16	yes		
	d) MB296B/Shi	12	no		
	e) MB320C/Shi	11	yes		
Figure 5E	a) Empty split-gal4/Trp	12	yes	one-sample t-test against zero; unpaired t-test	one-sample t-test:<br>a) 0.4322<br>b) 0.0048<br>c) 0.8050<br>d) 0.6081<br><br>unpaired t-test:<br>a) v. b) 0.0085<br>c) v. d) 0.5643
	b) MB630B/Trp	12	yes		
	c) Empty split-gal4/Trp	9	no		
	d) MB630B/Trp (mock)	9	yes		
Figure 5F	a) no reminder control	8	yes	one-sample t-test against zero;<br>ANOVA+Tukey;	one-sample t-test:<br>a) 0.1353<br>b) 0.3706<br>c) 0.33382<br><br>ANOVA+Tukey:<br>ANOVA: p=0.8958<br>a) v. b) 0.9768<br>a) v. c) 0.8864<br>b) v. c) 0.9627
	b) CS- as reminders in testing chamber	8	no		
	c) CS- as reminders in shock chamber	8	yes		
Figure 5G	a) no reminder control	8	yes	one-sample t-test against zero;<br>ANOVA+Tukey;	one-sample t-test:<br>a) 0.0627<br>b) 0.0559<br>c) 0.0023<br><br>ANOVA: 0.0025<br>a) v b) 0.9851<br>a) v c) 0.0075<br>b) v c) 0.0051
	b) react in plastic chamber	8	yes		
	c) react in the shock chamber	8	yes		
	a) paired odor v. unpaired odor	16	yes		one-sample t-test:<br>a) 0.4723

Extended Data Figure 1A	b) novel odor v. unpaired odor	16	yes	one-sample t-test against zero; ANOVA	b) 0.7459<br>c) 0.8180<br><br>ANOVA: 0.8354
	c) paired odor v. novel odor	16	yes		
Extended Data Figure 1B	a) 60V training	16	yes	one-sample t-test against zero; unpaired t-test	one-sample t-test:<br>a) 0.0027<br>b) 0.2275<br><br>unpaired t-test:<br>a) v b) 0.0426
	b) 60V retraining	16	yes		
Extended Data Figure 1C	a) immediate (trained group)	12	yes	one-sample t-test against zero; unpaired t-test	one-sample t-test:<br>a) <0.0001<br>b) <0.0001<br>c) 0.3646<br>d) 0.0001<br>e) 0.0001<br>f) 0.0018<br>unpaired t-test:<br>a) v. d): 0.5504<br>b) v. e) 0.0359<br>c) v. f) 0.0991
	b) 1h	12	no		
	c) 24h	12	yes		
	d) immediate after reminders (reminder group)	12	yes		
	e) 1h	12	yes		
	f) 24h	12	yes		
Extended Data Figure 1D	a) no reminder control	12	yes	one-sample t-test against zero; ANOVA+Tukey;	one-sample t-test:<br>a) 0.5764<br>b) 0.1156<br>c) 0.0045<br><br>ANOVA+Tukey:<br>ANOVA p=0.0016<br>a) v. b) 0.5399<br>a) v. c) 0.0233<br>b) v. c) 0.0014
	b) reminders in different context	12	yes		
	c) reminders in the same context	12	yes		
Extended Data Figure 2A	a) CS/Shi	12	yes	one-sample t-test against zero; ANOVA+Tukey;	one-sample t-test:<br>a) 0.0026<br>b) 0.0049<br>c) 0.0040<br><br>ANOVA+Tukey<br>ANOVA p=0.2009<br>a) v. b) 0.9937<br>a) v. c) 0.2765<br>b) v. c) 0.2346
	b) R13F02/CS	12	yes		
	c) R13F02/Shi	10	yes		
Extended Data Figure 2B	a) CS/RICIN	10	Yes	one-sample t-test against zero; ANOVA+Tukey;	one-sample t-test<br>a) < 0.0001<br>b) < 0.0001<br>c) 0.2529<br><br>ANOVA: < 0.0001
	b) R13F02/CS	11	Yes		
	c) R13F02/RICIN	11	yes		

					a) v b) 0.0315<br>a) v c) <0.0001<br>b) v c) 0.0369
Extended Data Figure 2C	a) Empty split-gal4/Shi	12	Yes	one-sample t-test against zero; unpaired t-test	one-sample t-test:<br>a) 0.0001<br>b) 0.0026<br>unpaired t-test:<br>a) v b) 0.0143
	b) NP1004/Shi	13	yes		
Extended Data Figure 2D	a1) Empty split-gal4/Shi	10	Yes	one-sample t-test against zero; unpaired t-test	one-sample t-test:<br>a1) 0.0001<br>b1) 0.0527<br>unpaired t-test:<br>a1) v b1) 0.0002<br><br>one-sample t-test:<br>a2) 0.0025<br>b2) 0.0401<br>c2) 0.0040<br>d2) 0.1500<br>unpaired t-test:<br>a2) v. b2) 0.8634<br>a2) v. c2) 0.1936<br>a2) v. d2) 0.1868<br><br>one-sample t-test:<br>a3) 0.0398<br>b3) 0.4883<br>c3) 0.0894<br>d3) 0.0104<br>unpaired t-test:<br>a3) v. b3) 0.1677<br>a3) v. c3) 0.5643<br>a3) v. d3) 0.6982
	b1) LH989/Shi	10	yes		
	a2) empty split-gal4/shi	8	yes		
	b2) LH1983/Shi	10	yes		
	c2) LH728/Shi	9	yes		
	d2) LH1293/Shi	10	no		
	a3) empty split-gal4/shi	8	yes		
	b3) LH1538/Shi	10	no		
	c3) LH291/Shi	10	no		
	d3) LH2193/Shi	10	yes		
Extended Data Figure 2F	a) Empty gal4/Shi	8	yes	one sample t-test against zero;<br>unpaired t-test	one-sample t-test:<br>a) 0.0047<br>b) 0.0006<br>unpaired t-test:<br>a) v. b) 0.4315
	b) R58E02/Shi	8	yes		
Extended Data Figure 2G	a) Empty split-gal4/Shi	23	yes	one-sample t-test against zero; unpaired t-test	one-sample t-test:<br>a) <0.0001<br>b) 0.4994<br>c) 0.0022<br>d) 0.0024<br>e) 0.0007<br>unpaired t-test:<br>a) v. b): 0.0023<br>a) v. c): 0.6795
	b) MB058B/Shi	19	no		
	c) MB320C/Shi	8	yes		
	d) MB630B/Shi	8	yes		
	e) MB296B/Shi	10	yes		

					a) v. d): 0.9942<br>a) v. e) 0.9756
Extended Data Figure 2H	a) Empty split-gal4/Shi	8	yes	one-sample t-test against zero	one-sample t-test:<br>a) 0.0134<br>b) 0.0020<br><br>unpaired t-test:<br>a) v b) 0.5093
	b) MB058/Shi	8	yes		
Extended Data Figure 2I	a) Empty split-gal4/Shi	9	yes	one sample t-test against zero;<br>unpaired t-test	a) 0.7268<br>b) 0.3212
	b) MB058/Shi	9	yes		
Extended Data Figure 2J	a) R58E02/Trp	6	not applicable	one-sample t-test; unpaired t-test	one-sample t-test:<br>a) <0.0001<br>b) 0.0225<br>c) 0.1093<br>d) < 0.0001<br>e) 0.0011<br><br>unpaired t-test:<br>a) v. c) <0.0001<br>b) v. c) 0.6297<br>d) v. c) 0.0030<br>e) v. c) 0.0120
	b) MB058B/Trp	5	not applicable		
	c) CS/Trp	5	not applicable		
	d) MB296B/Trp	6	not applicable		
	e) TH/Trp	5	not applicable		
Extended Data Figure 2K – first plot	a) MB058B/Chrimson + Light on, 0h test	17	yes	one-sample t-test against zero; unpaired t-test;	one-sample t-test:<br>a) 0.0001<br>b) <0.0001<br>c) 0.0087<br>d) 0.1087<br>unpaired t-test:<br>a) vs c) 0.0273<br>b) vs d) 0.0102
	b) Empty-gal4/Chrimson + Light on, 0h test	17	yes		
	c) MB058B/Chrimson + Light on, 1.5h test	17	yes		
	d) Empty-gal4/Chrimson + Light on, 1.5h test	17	yes		
Extended Data Figure 2K – second plot	a) MB058B/Chrimson + Light on	16	yes	one-sample t-test against zero; one-way ANOVA	one-sample t-test:<br>a) 0.0236<br>b) 0.0002<br>c) 0.0435<br>d) 0.0039
	b) Empty-gal4/Chrimson + Light on	16	yes		
	c) MB058B/Chrimson + no light	16	yes		

	d) Empty-gal4/Chrimson + no light	16	yes		One way ANOVA<br>a) vs b) vs c) vs d)<br>0.9996
Extended Data Figure 2L	a) MB058B-gal4/trp – no reminder	11	yes	one-sample t-test against zero; unpaired t-test	one-sample t-test:<br>a) 0.4885<br>b) 0.9562<br>unpaired t-test:<br>a) vs b) 0.6968
	b) MB058B-gal4/trp – unpaired odor	9	yes		
Extended Data Figure 3A	a) empty split-gal4/Shi	40	yes	one sample t-test against zero;<br>unpaired t-test but only with control data points that were done in parallel	one-sample t-test:<br>a) 0.0000<br>b) 0.0179<br>c) 0.0035<br>d) 0.0005<br>unpaired t-test:<br>a) v. b) 0.4336<br>a) v. c) 0.1934<br>a) v. d) 0.1978
	b) MB018B/Shi	8	yes		
	c) MB062B/Shi	8	yes		
	d) MB085C/Shi	12	yes		
Extended Data Figure 3B	a) Empty split-gal4/Shi	12	yes	one-sample t-test; unpaired t-test	one-sample t-test:<br>a) 0.0004<br>b) 0.0164<br><br>unpaired t-test:<br>a) v. b) 0.9080
	b) MB080C/Shi	12	yes		
Extended Data Figure 3C	a) Empty split-gal4/Shi	14	yes	one-sample t-test	one-sample t-test:<br>a) 0.6631<br>b) 0.7413
	b) MB080C/Shi	14	yes		
Extended Data Figure 3D	a) no reminder control	12	yes	one-sample t-test against zero; unpaired t-test	one-sample t-test:<br>a) 0.6355<br>b) <0.0001<br>unpaired t-test:<br>a) v b) 0.0003
	b) CS+ as reminders at 6h	12	yes		
Extended Data Figure 3E	a1) CS + trained	12	yes	Wilcoxon test	a1) v. b1) 0.1514<br><br>a2) v. b2) 0.5417
	b1) CS- trained	12	no		
	a2) CS + mock	13	no		
	b2) CS – mock	13	no		
Extended Data Figure 3F	a1) reminder trained	10	no	Kruskal Wallis; Wilcoxon test	Kruskal Wallis:<br>a1) v. a2) 0.2885<br><br>Wilcoxon test:<br>a1) v. a3) 0.9219<br>a2) v. a4) 0.7344<br><br>b1) v. b2) 0.3750<br>c1) v. c2) 0.2031
	a2) reminder mock	9	no		
	a3) test trained	10	no		
	a4) test mock	9	no		
	b1) CS+ trained	10	no		
	b2) CS- trained	10	no		
	c1) CS+ mock	9	no		
	c2) CS- mock	9	no		
	a1) CS+ trained	9	no	Kruskal Wallis	a1) v. a2) 0.8946

Extended Data Figure 3G	a2) CS+ mock	9	no		
Extended Data Figure 3H	a) control	8	yes	one-sample t-test; unpaired t-test	one-sample t-test:<br>a) 0.6400<br>b) 0.8637<br><br>unpaired t-test:<br>a) v b) 0.8708
	b) MB080C/Shi	8	yes		
Extended Data Figure 4A	a) CS+ mock	8	no	Wilcoxon test	Wilcoxon test:<br>a) v. b) 0.4609
	b) CS- mock	8	yes		
Extended Data Figure 4B	a) CS+ trained	11	no	Kruskal-Wallis	a) v. b) 0.7180
	b) CS+ mock	11	yes		
Extended Data Figure 4C	a) CS+	11	no	Wilcoxon test	Wilcoxon test:<br>a) v. b) 0.2783
	b) CS-	11	no		
Extended Data Figure 4D	a) CS+ trained	12	yes	Wilcoxon test	Wilcoxon test<br>a) v. b) 0.4213
	b) CS- trained	12	yes		
Extended Data Figure 4E	a) CS+ mock	12	no	Wilcoxon test	Wilcoxon test<br>a) v. b) 0.4238
	b) CS- mock	12	yes		
Extended Data Figure 5A	a) no reminder control	20	yes	one-sample t-test against zero; ANOVA+Tukey;	one-sample t-test:<br>a) 0.0659<br>b) 0.0008<br>c) 0.0886<br><br>ANOVA+Tukey:<br>ANOVA p=0.0097<br>a) v. b) 0.0128<br>a) v. c) 0.9352<br>b) v. c) 0.0417
	b) CS+ as reminders	19	no		
	c) novel odor as reminders	17	yes		
Extended Data Figure 5B	a) no reminder control	12	yes	one-sample t-test against zero; ANOVA+Tukey;	one-sample t-test:<br>a) 0.6210<br>b) 0.0586<br>c) <0.0001<br>d) 0.0131<br><br>ANOVA+Tukey:<br>ANOVA p<0.0001<br>a) v. b) 0.7518<br>a) v. c) 0.0025<br>a) v. d) 0.0077
	b) only context as reminders (no odor)	8	yes		
	c) CS+ as reminders	12	yes		
	d) CS- as reminders	12	yes		
	a) CS/Shi	8	no	one-sample t-test against	one-sample t-test:<br>a) 0.0043
	b) TH-GAL4/CS	9	yes		

Extended Data Figure 5C	c) TH-GAL4/Shi	9	yes	zero; ANOVA+Tukey;	b) 0.0007<br>c) 0.5050<br><br>ANOVA+Tukey<br>ANOVA p=0.0003<br>a) v. b) 0.6309<br>a) v. c) 0.0047<br>b) v. c) 0.0003
Extended Data Figure 5D	a) CS/Shi<br>b) TH/CS<br>c) TH/Shi	9<br>9<br>9	yes<br>yes<br>yes	one-sample t-test against zero; ANOVA+Tukey;	one-sample t-test:<br>a) <0.0001<br>b) 0.0021<br>c) 0.0014<br><br>ANOVA+Tukey:<br>ANOVA p=0.4061<br>a) v. b) 0.6095<br>a) v. c) 0.3956<br>b) v. c) 0.9289
Extended Data Figure 5E	a) empty split-gal4/shi<br>b) MB630B/shi	8<br>8	yes<br>yes	one-sample t-test; unpaired t-test	one-sample t-test:<br>a) 0.0079<br>b) 0.0001<br><br>unpaired t-test:<br>a) v. b) 0.4660
Extended Data Figure 5F	a) Empty split-gal4/Trp<br>b) MB630B/Trp	8<br>8	yes<br>yes	one-sample t-test against zero; unpaired t-test	one-sample t-test:<br>a) 0.3392<br>b) 0.0652<br><br>unpaired t-test:<br>a) v. b) 0.0636
Extended Data Figure 5G	a) empty split-gal4/Trp<br>b) MB058B/Trp	15<br>16	yes<br>yes	one-sample t-test; unpaired t-test	one-sample t-test:<br>a) 0.3466<br>b) 0.1353<br><br>unpaired t-test:<br>a) v. b) 0.0792
Extended Data Figure 5H	a) normal training (control)<br>b) transferred after CS+	8<br>8	yes<br>yes	one-sample t-test against zero; unpaired t-test	one-sample t-test:<br>a) 0.0016<br>b) 0.0007<br><br>unpaired t-test:<br>0.5156
Extended Data Figure 5I	a) normal training (control)<br>b) light off for CS-	12<br>12	yes<br>yes	one-sample t-test; unpaired t-test	one-sample t-test:<br>a) 0.0080<br>b) 0.0228

					unpaired t-test: 0.6006
Extended Data Figure 5J	a) normal training (control)	8	yes	one-sample t-test; unpaired t-test	one-sample t-test:<br>a) 0.0203<br>b) 0.0315<br><br>unpaired t-test:<br>a) v. b) 0.5238
	b) first CS-, then CS+ in the training	8	yes		

Supplementary Table 3: Statistical analyses associated with all figures. The table includes sample size, normality testing, statistical tests applied and post hoc analyses for each experiment and figure panel
